# Ligand-Enabled Copper-Mediated Radioiodination of Arenes

**DOI:** 10.1021/acs.orglett.4c00356

**Published:** 2024-02-09

**Authors:** Holly McErlain, Matthew J. Andrews, Allan J. B. Watson, Sally L. Pimlott, Andrew Sutherland

**Affiliations:** † School of Chemistry, University of Glasgow, Glasgow, G12 8QQ, U.K.; ‡ EaStCHEM, School of Chemistry, University of St Andrews, North Haugh, St Andrews, Fife KY16 9ST, U.K.; § West of Scotland PET Centre, Greater Glasgow and Clyde NHS Trust, Glasgow, G12 OYN, U.K.

## Abstract

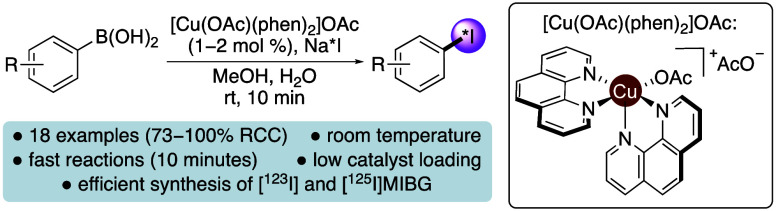

The discovery of a copper precatalyst that facilitates the key mechanistic steps of arene halodeboronation has allowed a step change in the synthesis of radioiodine-containing arenes. The active precatalyst [Cu(OAc)(phen)_2_]OAc was shown to perform room temperature radio-iododeboronation of aryl boronic acids with 1–2 mol % loadings and 10 min reaction times. These mild conditions enable particularly clean reactions, as demonstrated with the efficient preparation of the radiopharmaceutical and SPECT tracer, *meta*-iodobenzylguanidine (MIBG).

Organic compounds labeled with radioactive isotopes of iodine are widely used as tools for diagnostic imaging and radiotherapy in nuclear medicine and for radioassays in biomedical research and drug discovery.^1,2^ Compounds labeled with iodine-131 are employed as radiopharmaceuticals for radiotherapy,^3^ while iodine-125 derivatives are used for preclinical *in vitro* measurements in biomedical studies.^4^ The other commonly used radioisotope is iodine-123, which, in combination with single photon emission computed tomography (SPECT), is used for *in vivo* diagnostic imaging of disease.^1,2^ Important SPECT imaging agents include the commercially available radiopharmaceutical, [^123^I]MIBG (**1**),^5^ which is used for the identification of primary tumors and metastatic sites associated with neuroblastoma, and [^123^I]iomazenil (**2**),^6^ a SPECT tracer of central-type benzodiazepine receptors in brain tissue (Figure 1a).

**Figure 1:**
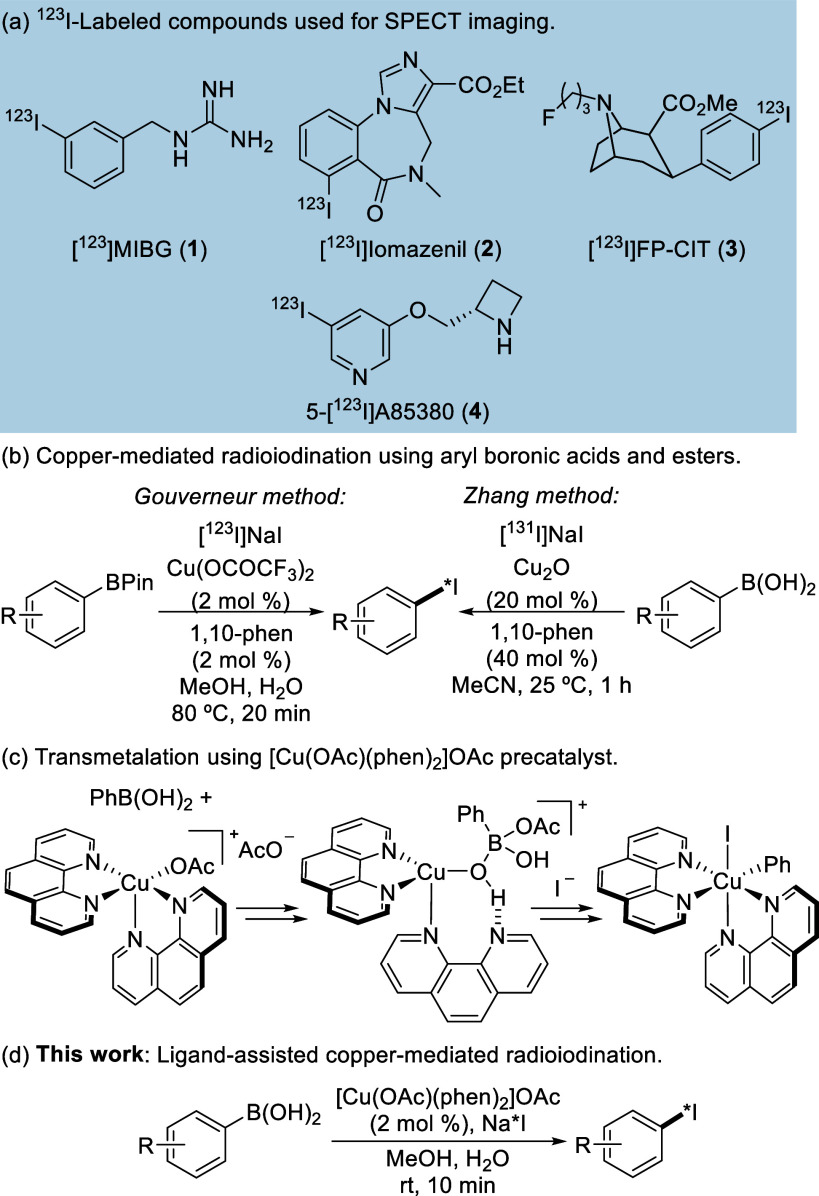
(a) SPECT imaging agents. (b) Cu(II)-mediated radio-iodination of aryl Bpin esters and aryl boronic acids. (c) Transmetalation of aryl boronic acids using [Cu(OAc)(phen)_2_]OAc. (d) This work.

Due to the relatively stable Csp^2^–I bond, radioiodine is commonly incorporated within an arene moiety. Traditionally, this was accomplished by isotopic exchange via S_N_Ar reactions or electrophilic aromatic substitution methods such as iodo-destannylation of organotin intermediates.^7^ However, the harsh conditions of these approaches and the toxicity associated with organotin compounds have resulted in the recent development of novel methods for the radioiodination of arenes that can be performed from nontoxic precursors or using mild conditions. These include the click-type reaction of azides and alkynes in the presence of [^125^I]iodide,^8^ a Sandmeyer radioiodination of diazonium salts,^9^ and the use of silver(I)-based Lewis acids for the radioiodination of electron-rich arenes.^10^ Methods using transition metals have also been reported, such as a nickel-mediated radioiodination of aryl bromides,^11^ arene C–H radioiodination using palladium acetate,^12^ and radio-iododecarboxylation of aryl carboxylic acids using gold(I) intermediates.^13^ The widespread availability of aryl boronic acids and esters has meant that these have also been investigated as precursors for transition-metal-mediated radioiodination methods.^14^ In particular, several copper-mediated radioiodination reactions of aryl boron compounds have been reported. In 2016, Gouverneur and co-workers described the copper(II)-catalyzed radioiodination of aryl boronic acid pinacol esters (Figure 1b) via a proposed Chan–Lam mechanism.^15^ With a loading of 2 mol % and a reaction temperature of 80 °C, iodine-123 labeling of a broad range of substrates was complete after 20 min reaction times. Concurrent work by the Zhang group reported room temperature iodine-131 labeling of aryl boronic acids (Figure 1b).^16^ Using copper(I) oxide (20 mol %), the radioiodinations were complete after 1 h. In both methods, 1,10-phenathroline was used as the ligand. Subsequent work by the Mach group described the room temperature radioiodination of aryl boronic acid pinacol esters using Cu(pyridine)_4_(OTf)_2_ (5 mol %) as the catalyst and phenathroline ligands for the preparation of ^125^I-labeled PARP inhibitors.^17^

We have been intrigued by the combination of copper complexes and 1,10-phenanthroline ligands for Chan–Lam halodeboronation of aryl boron compounds. Some of us recently reported a detailed experimental and computational investigation of the copper-catalyzed iododeboronation mechanism.^18^ Using Cu(OAc)_2_ and 1,10-phenanthroline, this study identified [Cu(OAc)(phen)_2_]OAc as the reaction precatalyst that was found to be critical for key steps of the catalytic cycle, including transmetalation via hydrogen bonding to the boronate (Figure 1c) and oxidative events such as disproportionation and turnover. Following the discovery of [Cu(OAc)(phen)_2_]OAc as an active precatalyst for halodeboronation, we were interested in determining whether the use of this complex could overcome the limitations of previous key radioiodination methods with aryl boronates. Although the Gouverneur method (Figure 1b) used a low copper loading (2 mol %), high temperatures (80 °C) were required.^15^ The Zhang method was conducted at room temperature but used high loadings of both catalyst (20 mol %) and ligand (40 mol %).^16^ We wished to establish whether the precatalyst could be used at low loading and at room temperature while maintaining fast reaction times. Herein, we report a step change in the ability to perform radioiodination of aryl boronic acids using [Cu(OAc)(phen)_2_]OAc. We describe the efficient and clean radioiodination of aryl boronic acid substrates at room temperature using 1–2 mol % loadings after 10 min reaction times (Figure 1d). We also demonstrate the compatibility of the copper(II) precatalyst with amine functional groups in a final-step synthesis of SPECT tracer [^123^I]MIBG (**1**) from an unprotected precursor.

The study began with the investigation of [Cu(OAc)(phen)_2_]OAc-catalyzed radioiodination of 4-methoxyphenyl boronic acid (**5a**) using no-carrier added [^125^I]NaI (Table 1). The active precatalyst [Cu(OAc)(phen)_2_]OAc was readily prepared from copper(II) acetate and 1,10-phenanthroline under basic conditions and is both air and moisture stable (see Supporting Information for preparation). It should be noted that iodine-125 was used as the longer half-life (*t*_1/2_ ∼ 59.4 days) of this isotope is readily amenable to optimization studies. An initial reaction was performed at 10 mol % catalyst loading (relative to the boronic acid substrate) and at a temperature of 50 °C (entry 1). After a reaction time of 20 min, a radiochemical conversion (RCC) of 100% was observed.^19^ Subsequent reactions showed that the temperature and catalyst loading could be reduced to 20 °C and 2 mol %, respectively, while maintaining a 20 min reaction time and 100% RCC (entries 2–4). SPECT imaging agents are typically prepared in low micromole quantities, and thus, the scalability of this method was examined (entry 5). A 10-fold reduction of reaction scale, from 6.6 to 0.66 μmol of boronic acid substrate again gave 100% RCC. At this scale and these conditions, the reaction time could be reduced further to 10 min (entry 6); however, reduction of the catalyst loading to 1 mol % resulted in a slight drop of RCC to 89% (entry 7). Finally, to explore the effect of outer sphere anion displacement during the radioiodination reaction, the acetate anion was exchanged to chloride to form the [Cu(phen)_2_(OAc)]Cl complex (entry 8).^18^ Although radioiodination was achieved at room temperature and low catalyst loading with the chloride catalyst, a 1 h reaction time was required to achieve a similar RCC as the [Cu(OAc)(phen)_2_]OAc catalyst. Overall, the optimization study revealed that the active precatalyst could overcome the high temperatures or high catalyst loading of previous methods,^15,16^ allowing radioiodination using 1–2 mol % loadings at room temperature and reaction times of only 10 min (entries 6 and 7). Furthermore, as exemplified by the radio-HPLC chromatogram of the crude reaction mixture (Figure 2), the use of the [Cu(OAc)(phen)_2_]OAc precatalyst facilitates a particularly clean radioiodination reaction with no other radiolabeled side-products present. During the course of this study, similar reaction profiles were observed for the majority of substrates investigated, generating radioiodinated products more cleanly from aryl boronic acids than other methods.^14−17^

**Figure 2:**
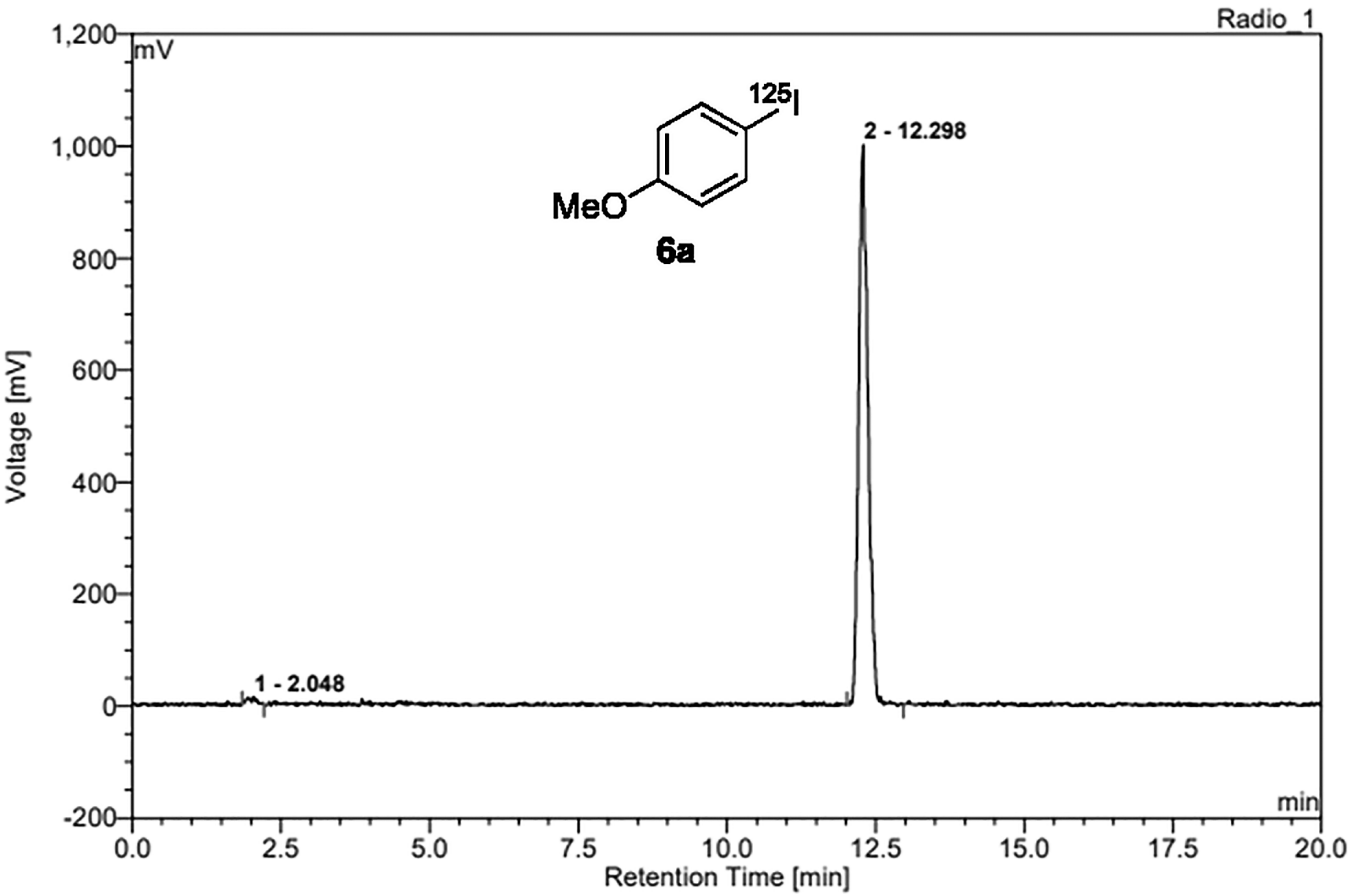
Analytical radio-HPLC trace of the crude reaction mixture from radioiodination of **5a**, showing 99% RCC.

**Table 1:**
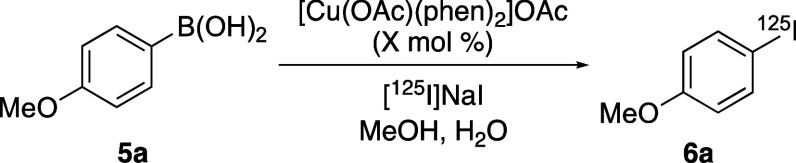
Optimization Studies for the Copper(II)-Mediated Radioiodination of **5a**

entry	catalyst loading (mol %)	temp (°C)	time (min)	RCC (%)^a^
1	10	50	20	100
2	10	30	20	100
3	10	20	20	100
4	2	20	20	100
5^b^	2	20	20	100
6^b^	2	20	10	99
7^b^	1	20	10	89
8^b^^,^^c^	2	20	60	99

a Radiochemical conversions (RCC) were determined by radio-HPLC of crude reaction mixtures. Product identity was confirmed by HPLC using 4-iodoanisole as the reference standard.

b Amount of substrate was reduced from 6.6 to 0.6 μmol.

c Conducted using [Cu(phen)_2_(OAc)]Cl.

Using the optimized conditions, we then explored the scope of the reaction (Scheme 1a). In general, radioiodination of aryl boronic acids (**5a**–**5m**) with electron-rich or electron-deficient functional groups and various substitution patterns reacted cleanly under the optimized conditions to give ^125^I-labeled products in 86–100% RCC. Only amine- and carboxylic acid-substituted phenyl boronic acids **5c** and **5k** required longer reactions or higher temperatures. Despite this, **6c** and **6k** were both formed in 74% RCC. The optimized reaction conditions were also applicable to alkenyl (**5n**) and heteroaryl (**5o** and **5p**) boronic acid substrates and gave the ^125^I-labeled products in excellent RCC (92–97%). This part of the study again reinforced the advantage of using the copper precatalyst, with nearly all reactions performed at room temperature, using only 2 mol % loading and typically requiring 10 min reaction times. For example, the Gouverneur method, at 80 °C, generated phenol **6b** and 3-nitrophenyl **6g** in lower RCCs (39% and 63%, respectively), from the corresponding boronic acids.^15^ The efficiency of our method is similar to the Zhang study yet is much faster (10 min versus 1 h), with 10-fold lower catalyst loadings (2 versus 20 mol %).^16^ In our study, the only substrate requiring an elevated reaction temperature (60 °C) was 4-aminophenyl boronic acid (**5c**). Interestingly, unprotected primary aniline substrates are absent from previous copper-mediated radioiodination reactions,^15−17^ indicating that unlike other copper catalyst and ligand combinations, the active precatalyst can still perform radioiodination without issues associated with amino-group coordination.

**Scheme 1:**
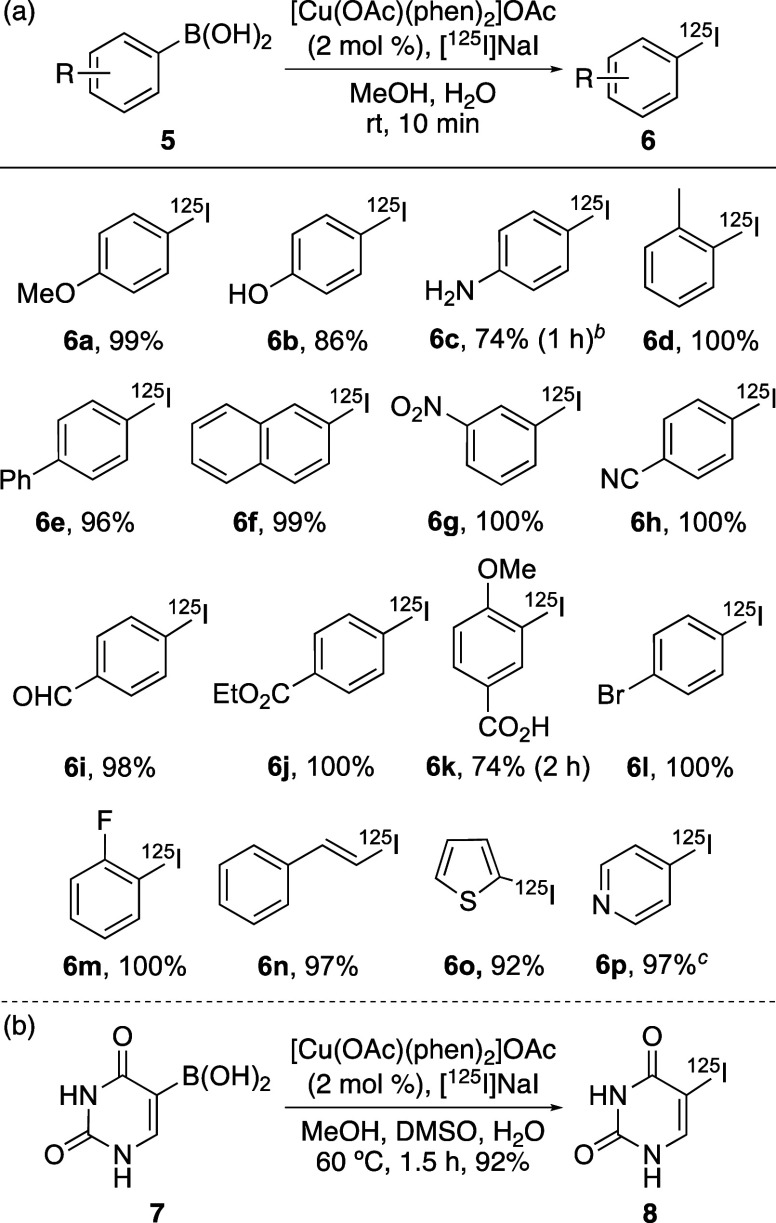
Substrate Scope of [Cu(OAc)(phen)_2_]OAc-Mediated Radioiodination Reaction RCCs by radio-HPLC of the crude reaction mixture. Reaction conducted at 60 °C. MeCN was used as a cosolvent.

The use of this method for the synthesis of biologically active targets was also examined. The synthesis of iodouracil, a precursor of uridine-based SPECT imaging agents,^20^ and an inhibitor of the anticancer target, dihydropyrimidine dehydrogenase, was investigated.^21^ Initial solubility issues with uracil-5-boronic acid (**7**) were overcome using methanol, DMSO, and water as cosolvents (Scheme 1b). Radioiodination of **7** under these conditions was achieved using low catalyst loading (2 mol %), and while a temperature of 60 °C and reaction time of 1.5 h were required, this gave [^125^I]iodouracil (**8**) in 92% RCC.

Following exploration of the substrate scope, application of the ligand-assisted copper-catalyzed radioiodination for the preparation of a SPECT imaging agent was investigated. *meta*-Iodobenzylguanidine (MIBG) is a structural mimic of norepinephrine that is taken up via an active mechanism into neuroendocrine cells. This results in the selective accumulation of MIBG in neuroectodermally derived tumors such as neuroblastoma, carcinoids, and medullary carcinoma of the thyroid.^22^ For this reason, radiolabeled MIBG has been developed as a clinic-based radiopharmaceutical. In ^123^I-form, MIBG is used for diagnosis, while labeled with iodine-125 or iodine-131, MIBG has found widespread application for imaging and therapy of neuroblastoma and other neural crest tumors.^5,23^ The importance of radioiodinated MIBG has resulted in a variety of synthetic approaches, including solid state halogen exchange and electrophilic iodination from organosilane or organostannane precursors.^7,24^ To evaluate whether ligand-assisted copper-catalyzed radioiodination would allow the effective synthesis of radiolabeled MIBG, a suitable boronic acid precursor was prepared (Scheme 2). Under basic conditions, 3-(aminomethyl)benzeneboronic acid **9** was reacted with commercially available Boc-protected 1*H*-pyrazole-1-carboxamide **10**, which gave coupled product **11** in 76% yield. Recent syntheses of radiolabeled MIBG with this precursor have performed the radioiodination step first, followed by acid-mediated removal of the Boc-protecting groups.^14−16^ To allow direct comparison, we performed the same two-step approach. Thus, [Cu(OAc)(phen)_2_]OAc-catalyzed radioiodination of precursor **11** using the optimized conditions was investigated. Using standard catalyst loading (2 mol %), the reaction was complete after 10 min at room temperature. Deprotection using 6 M HCl with a reaction time of 10 min gave [^125^I]MIBG (**13**) in 95% RCC over the two steps. Compared to base-, gold-, and other copper-mediated radio-iododeboronations,^14−16^ this represents one of the most rapid, clean, and efficient syntheses of radiolabeled MIBG, while using a low catalyst loading under mild conditions. Although a short, final step deprotection is not an issue for relatively long-lived radioiodine isotopes, it is still preferable to introduce a radiolabel at the end of a synthesis. This is to maximize utilization of the expensive radioisotope and minimize handling of radioactive material. For these reasons, we proposed an alternative synthesis of [^125^I]MIBG (**13**) involving deprotection and then radioiodination. As an aniline boronic acid was successfully radioiodinated during the substrate screen, we were confident that the precatalyst would accommodate the unprotected guanidine without coordination issues. Therefore, guanidine **11** was treated with 6 M HCl, which gave precursor **14** in 93% yield. Ligand-assisted copper-catalyzed radioiodination (2 mol %) at room temperature required a longer reaction time of 25 min but resulted in the clean, final-step synthesis of [^125^I]MIBG (**13**) in 96% RCC. Thus, the copper(II)-precatalyst was compatible with the unprotected guanidine moiety.

**Scheme 2:**
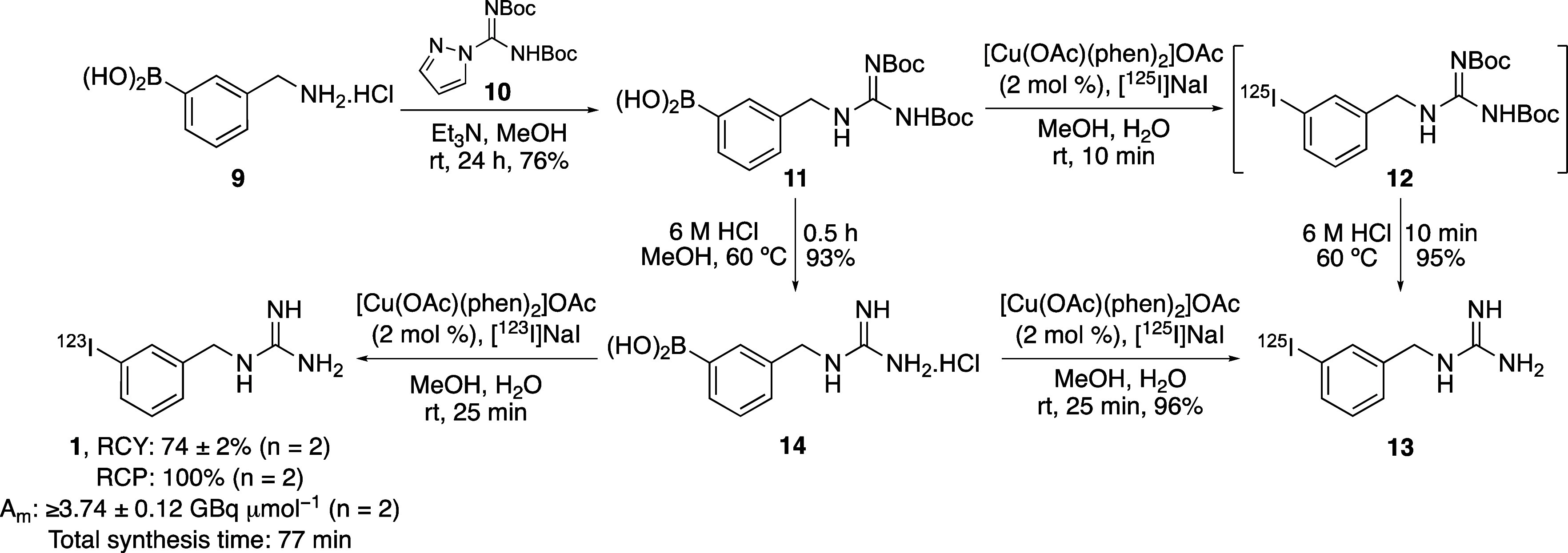
Radiosynthesis of [^125^I]MIBG (**13**) and [^123^I]MIBG (**1**)

This final-step radioiodination strategy was also employed for the synthesis of the SPECT imaging agent [^123^I]MIBG (**1**) (Scheme 2). Using no-carrier added [^123^I]iodide (27.6–28.9 MBq, *t*_1/2_ = 13.2 h) and the optimized conditions, the room temperature radioiodination of **14** was again complete in 25 min. Following purification by HPLC and formulation, [^123^I]MIBG (**1**) was isolated in 74% radiochemical yield (RCY), 100% radiochemical purity (RCP), and molar activity (A_m_) of ≥3.74 GBq μmol^–1^. These results compare favorably to previous no-carrier added methods for the radiosynthesis of MIBG,^24^ allowing the preparation of this radiopharmaceutical via a fast reaction and low catalyst loading in excellent RCY. In addition to these advances, this approach avoids the use of strong oxidizing conditions and toxic organotin precursors.

In summary, the discovery of a copper(II)-precatalyst that can perform highly effective, ligand-assisted halodeboronation has allowed a step change in the preparation of radioiodinated arenes. Compatible with a wide range of readily available (hetero)aryl and alkenyl boronic acids, the precatalyst was found to perform rapid radio-iododeboronation reactions while avoiding high temperatures and high catalyst loadings. Using only 1–2 mol % of [Cu(OAc)(phen)_2_]OAc at room temperature permitted the clean production of radiolabeled products with excellent RCCs after only 10 min reaction times. The precatalyst was compatible with unprotected amines, allowing the radioiodination of an aniline substrate and efficient access to [^123^I]MIBG or [^125^I]MIBG from an unprotected guanidine precursor. The ability of this method to produce MIBG under these conditions demonstrates the potential of this copper(II)-precatalyst for the future development and production of other iodine-based radiopharmaceuticals.

## Data Availability

The data underlying this study are available in the published article and its Supporting Information.

## Supporting Information Available

Experimental procedures, characterization data, radio- and UV-HPLC traces, NMR spectra of all novel compounds (PDF)
