# Supplementary material for: Ligand-Enabled Copper-Mediated Radioiodination of Arenes
Source: Org Lett. 2024 Feb 9;26(7):1528–32. doi: 10.1021/acs.orglett.4c00356 (PMC10897930; doi:10.1021/acs.orglett.4c00356)

Supporting Information for:

Ligand-Enabled Copper-Mediated Radioiodination of Arenes

Holly McErlain,[†] Matthew J. Andrews,[‡] Allan J. B. Watson,[‡] Sally L. Pimlott[§]

and Andrew Sutherland[†]*

[†]School of Chemistry, University of Glasgow, Glasgow G12 8QQ, U.K. [‡]EaStCHEM, School of Chemistry, University of St Andrews, North Haugh, St Andrews, Fife, KY16 9ST, U.K. [§]West of Scotland PET Centre, Greater Glasgow and Clyde NHS Trust, Glasgow, G12 OYN, U.K.

Table of Contents

1. General Experimental S2

2. Experimental Procedures and Spectroscopic Data for all Compounds S2–S5

3. Radiochemistry Methodology S5–S14

4. Radio HPLC and UV-Vis HPLC Chromatograms S15–S26

5. References S27

6. ^{1}H and ^{13}C NMR Spectra for All Novel Compounds S28–S35

1. General Experimental

Boronic acid precursors and the corresponding iodine-127 authentic reference compounds were prepared as detailed below or purchased from commercial suppliers. (*E*)-2-Iodoethenylbenzene was prepared as reported in the literature.[1] Dry solvents were purified using a PureSolv 500 MD solvent purification system (acetonitrile) or purchased from commercial sources (methanol). All reactions were performed under an atmosphere of air unless stated otherwise. All reactions performed at elevated temperatures were heated using an oil bath. Glassware was dried in an oven at 140 °C for a minimum of 16 h, purged with argon, and cooled to room temperature. Brine refers to a saturated aqueous solution of sodium chloride. Merck aluminium-backed plates pre-coated with silica gel 60 (UV_{254}) were used for thin layer chromatography and were visualized under UV light (254/365 nm) and/or stained with potassium permanganate or ninhydrin solution. Flash column chromatography was carried out using Merck Geduran Si 60 silica gel (40–63 µm). ^{1}H and ^{13}C NMR spectra were recorded on a Bruker DPX 400, Bruker AVI 400, or Bruker AVIII 400 spectrometer, with chemical shift values reported in ppm relative to tetramethylsilane (δ_H 0.00 and δ_C 0.0), $CHCl_3$ and $CDCl_3$ (δ_H 7.26 and δ_C 77.2), CH_3OH and CD_3OD (δ_H 3.31 and δ_C 49.0), or DMSO and DMSO-d_6 (δ_H 2.50 and δ_C 39.5). Assignments of ^{13}C NMR signals are based on HMBC and DEPT experiments. Mass spectra were obtained using a JEOL JMS-700 spectrometer, a Bruker micrOTOF-Q spectrometer, or an Agilent 6125B spectrometer. Melting points were determined on a Gallenkamp, Stuart Scientific, or Reichert platform melting point apparatus and are uncorrected. Infrared spectra were recorded neat using a Shimadzu FTIR-8400S or a Shimadzu IRPrestage-21 spectrometer.

2. Experimental Procedures and Spectroscopic Data for all Compounds

{3-[2,3-Bis(*tert*-butoxycarbonyl)guanidino]methylphenyl}boronic acid (11)[2]

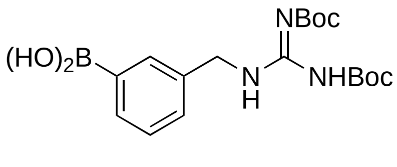

In an oven-dried flask under argon, *N*,*N*'-bis(*tert*-butoxycarbonyl)-1*H*-pyrazole-1-carboxamidine (**10**) (155 mg, 0.500 mmol) and 3-(aminomethyl)benzeneboronic acid hydrochloride (**9**) (103 mg, 0.550 mmol) were dissolved in anhydrous methanol (1.5 mL). Triethylamine (0.210 mL, 1.50 mmol) was added and the reaction mixture stirred at room temperature for 24 h. The solvent was removed *in vacuo*. Purification by flash column chromatography, eluting with 1–2% gradient of methanol in dichloromethane gave {3-[2,3-bis(*tert*-butoxycarbonyl)guanidino]methylphenyl}boronic acid (**11**) as

a white solid (149 mg, 76%). Mp 111–113 °C. Spectroscopic data were consistent with the literature.[2] ^{1}H NMR (400 MHz, CD_3OD) δ 7.75–7.49 (m, 2H), 7.38–7.28 (m, 2H), 4.55 (s, 2H), 1.50 (s, 9H), 1.47 (s, 9H); $^{13}C\{^{1}H\}$ NMR (101 MHz, CD_3OD) δ 164.5 (C), 157.5 (C), 154.2 (C), 138.0 (d, $^{3}J_{C\text{-}B}$ 31.5 Hz, C), 134.0 (d, $^{2}J_{C\text{-}B}$ 42.0 Hz, 2 × CH), 130.2 (d, $^{3}J_{C\text{-}B}$ 50.9 Hz, CH), 129.1 (CH), 84.5 (C), 80.4 (C), 45.5 (CH_2), 28.6 (3 × CH_3), 28.2 (3 × CH_3), (signal for carbon directly attached to boron is not observed); MS (ESI) *m/z* 394 (M + H^+, 20).

[3-(Guanidinomethyl)phenyl]boronic acid hydrochloride (14)

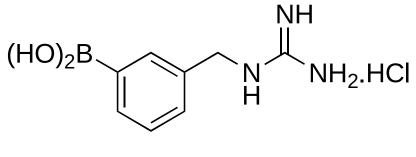

To a solution of {3-[2,3-bis(*tert*-butoxycarbonyl)guanidino]methylphenyl}boronic acid (**11**) (78.7 mg, 0.200 mmol) in methanol (3.33 mL) was added 6 M aqueous hydrochloric acid (3.33 mL, 20.0 mmol). The reaction mixture was heated to 60 °C and stirred for 0.5 h. The solvent was removed *in vacuo* and the crude material was azeotropically dried with toluene (3 × 3 mL). Toluene (3 mL) was added to the crude material and an off-white precipitate was formed by sonication of the solution. The supernatant was decanted to give [3-(guanidinomethyl)phenyl]boronic acid hydrochloride (**14**) as an off-white solid (42.6 mg, 93%). Mp 122–124 °C; IR (neat) 3302, 3171, 1671, 1640, 1434, 1352, 1320, 1072, 992 cm^{-1}; ^{1}H NMR (400 MHz, DMSO-d_6) δ 8.22–7.96 (m, 3H), 7.77–7.70 (m, 2H), 7.68–6.73 (m, 6H), 4.37 (d, J = 6.0 Hz, 2H); $^{13}C\{^{1}H\}$ NMR (101 MHz, DMSO-d_6) δ 157.0 (C), 136.0 (C), 134.6 (C), 133.2 (CH), 133.1 (CH), 129.0 (CH), 127.6 (CH), 44.2 (CH_2); MS (APCI) *m/z* 194 (M + H^+, 100); HRMS (APCI) *m/z*: $[M + H]^+$ Calcd for $C_8H_{12}BN_3O_2H$ 194.1097; Found 194.1095.

N′,*N″*-Bis(*tert*-butoxycarbonyl)-*N*-(3-iodobenzyl)guanidine (S1)[3]

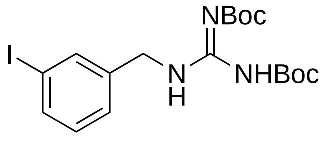

N,*N*’-Bis(*tert*-butoxycarbonyl)-1*H*-pyrazole-1-carboxamidine (**10**) (155 mg, 0.500 mmol) and 3-iodobenzylamine (128 mg, 0.550 mmol) were dissolved in chloroform (5 mL). Triethylamine (0.210 mL, 1.50 mmol) was added and the reaction mixture stirred at room temperature for 22 h. The mixture was diluted with chloroform (5 mL) and then washed with water (3 × 10 mL). The organic layer was dried ($MgSO_4$), filtered and concentrated *in vacuo*. Purification by flash column chromatography, eluting with 5% ethyl acetate in hexane gave *N′*,*N″*-bis(*tert*-butoxycarbonyl)-*N*-(3-

iodobenzyl)guanidine (**S1**) as a white solid (130 mg, 55%). Mp 104–106 °C. Spectroscopic data were consistent with the literature.[3] ^{1}H NMR (400 MHz, $CDCl_3$) δ 11.53 (s, 1H), 8.58 (s, 1H), 7.67 (s, 1H), 7.62 (d, J = 8.0 Hz, 1H), 7.28 (d, J = 7.6 Hz, 1H), 7.07 (dd, J = 8.0, 7.6 Hz, 1H), 4.57 (d, J = 5.6 Hz, 2H), 1.51 (s, 9H), 1.49 (s, 9H); ^{13}C{^{1}H} NMR (101 MHz, $CDCl_3$) δ 163.7 (C), 156.3 (C), 153.3 (C), 139.9 (C), 137.1 (CH), 136.8 (CH), 130.6 (CH), 127.2 (CH), 94.7 (C), 83.5 (C), 79.6 (C), 44.2 (CH_2), 28.4 (3 × CH_3), 28.2 (3 × CH_3); MS (ESI) *m/z* 476 (M + H^+, 100).

_N_-(3-Iodobenzyl)guanidine hydrochloride (S2)

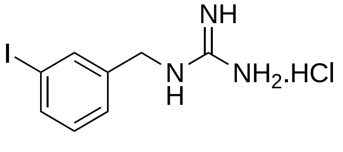

To a solution of *N′*,*N″*-bis(*tert*-butoxycarbonyl)-*N*-(3-iodobenzyl)guanidine (**S1**) (100 mg, 0.210 mmol) in methanol (3.50 mL) was added 6 M aqueous hydrochloric acid (3.50 mL, 21.0 mmol). The reaction mixture was heated to 60 °C and stirred for 0.5 h. The solvent was removed *in vacuo* and the crude material was azeotropically dried with toluene (5 × 3 mL) to give *N*-(3-iodobenzyl)guanidine hydrochloride (**S2**) as an off-white solid (54.3 mg, 83%). Mp 177–180 °C; IR (neat) 3306, 3143, 2919, 1606, 1591, 1420, 1344, 1063, 996 cm^{-1}; ^{1}H NMR (400 MHz, CD_3OD) δ 7.72 (s, 1H), 7.69 (d, J = 8.0 Hz, 1H), 7.34 (d, J = 7.6 Hz, 1H), 7.17 (dd, J = 8.0, 7.6 Hz, 1H), 4.38 (s, 2H); ^{13}C{^{1}H} NMR (101 MHz, CD_3OD) δ 157.3 (C), 138.8 (C), 136.8 (CH), 135.9 (CH), 130.3 (CH), 126.2 (CH), 93.8 (C), 43.7 (CH_2); MS (APCI) *m/z* 276 (M + H^+, 100); HRMS (APCI) *m/z*: $[M + H]^+$ Calcd for $C_8H_{10}IN_3H$ 275.9992; Found 275.9988.

(Acetato-*κO*)bis(1,10-phenathroline-*κ*^{2}*N*,*N'*)copper(II) acetate, [Cu(OAc)(phen)$_2$]OAc[4]

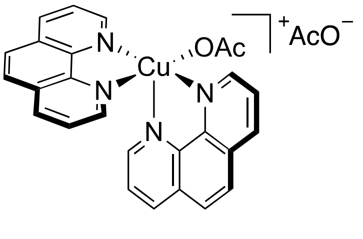

To a solution of cupric acetate monohydrate (0.299 g, 1.50 mmol) in methanol (10 mL) was added an aqueous solution of 1 M potassium hydroxide (3 mL, 3.0 mmol), followed by 1,10-phenanthroline (0.541 g, 3.0 mmol). The mixture was stirred overnight forming a dark green solution. This was evaporated to dryness under reduced pressure before acetone (10 mL) was added. This was concentrated under reduced pressure, before crystallization was induced through warming, resulting

in the precipitation of dark blue crystals. These were isolated by filtration, washed with acetone, and dried in air to give the desired complex (0.391 g, 48% yield). Data were consistent with the literature.[4]

3. Radiochemistry Methodology

3.1. General Experimental Information for Radioiodination with [^{125}I]NaI

Sodium [^{125}I]iodide was purchased from Perkin Elmer (product number NEZ033H005MC) as no-carrier added [^{125}I]NaI in 0.1 M sodium hydroxide (pH 12–14) aqueous solution with a concentration of either 12.92 GBq/mL (batch 1) or 13.77 GBq/mL (batch 2). Batch 1 was diluted with methanol:water (4:1) to give a total volume of 250 µL. Batch 2 was diluted with water to give a total volume of 250 µL. All radiochemical conversions were determined by radio-HPLC analysis of an aliquot from the reaction mixture.

3.2. Analytical Radio-HPLC Methods for Determination of Radiochemical Conversion

Analytical HPLC was performed with a Dionex Ultimate 3000 HPLC system equipped with a Flowstar LB 513 NaI scintillation detector with BGO-X cell, and a DAD-3000 UV detector using a Synergi 4 µm Hydro-RP 80 Å column (150 × 4.6 mm) with a 10 mm guard cartridge, UV 254 nm and flow 1 mL/min. The mobile phase for the analysis of substrates was 0.1% trifluoroacetic acid in water and 0.1% trifluoroacetic acid in acetonitrile. Analysis of the reaction mixture (to assess radiochemical conversion) was performed using one of the four methods shown below. Co-elution with the UV signal from the iodine-127 authentic reference compound was used to confirm the identity of the iodine-125 product from each reaction described below.

Analytical HPLC Method A:

Time (mins)	0.1% TFA in MeCN (%)	0.1% TFA in H_2O (%)
**0–15**	40–90	60–10
**15–17**	90	10
**17–18**	90–40	10–60
**18–20**	40	60

Analytical HPLC Method B:

Time (mins)	0.1% TFA in MeCN (%)	0.1% TFA in H_2O (%)
**0–15**	10–95	90–5
**15–17**	95	5
**17–18**	95–10	5–90
**18–20**	10	90

Analytical HPLC Method C:

Time (mins)	0.1% TFA in MeCN (%)	0.1% TFA in H_2O (%)
**0–10**	30	70

Analytical HPLC Method D:

Time (mins)	0.1% TFA in MeCN (%)	0.1% TFA in H_2O (%)
**0–2**	10	90
**2–10**	10–95	90–5
**10–12**	95	5
**12–13**	95–10	5–90
**13–15**	10	90

3.3. General Experimental Procedure for Radioiodination with $[^{125}I]NaI$

To a vial containing boronic acid (0.66 µmol) was added $[Cu(OAc)(phen)_2]OAc$ (7.2 µg, 0.013 µmol) in methanol:water (4:1, 100 µL) from a stock solution. The solution was then mixed by vortex for 30 seconds. To the reaction vessel was added $[^{125}I]NaI$ (1.5–2.9 MBq, 5 µL of batch 1 or 2 $[^{125}I]NaI$ solution). The reaction mixture was mixed by vortex for 5 seconds and then left at room temperature for 10 minutes. An aliquot (10 µL) of the reaction mixture was removed and diluted to a total volume of 500 µL using a 1:1 mixture of 0.1% trifluoroacetic acid in water and 0.1% trifluoroacetic acid in acetonitrile. An aliquot (20 µL) of this solution was then subjected to radio-HPLC analysis to determine the radiochemical conversion.

3.4. Radioiododeboronation Experimental Procedures and Characterisation Data

4-[^{125}I]Iodoanisole (6a)

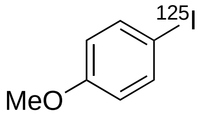

The reaction was carried out according to the general procedure as described in section 3.3 using 4-methoxyphenylboronic acid (**5a**) (0.10 mg, 0.66 µmol). The reaction was run at room temperature for 10 minutes. Analysis by radio-HPLC using method A gave a radiochemical conversion of 99%.

4-[^{125}I]Iodophenol (6b)

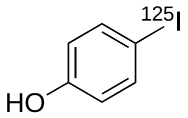

The reaction was carried out according to the general procedure as described in section 3.3 using 4-hydroxyphenylboronic acid (**5b**) (0.09 mg, 0.66 µmol). Analysis by radio-HPLC using method A gave a radiochemical conversion of 86%.

4-[^{125}I]Iodoaniline (6c)

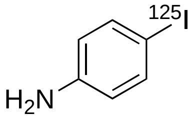

The reaction was carried out according to the general procedure as described in section 3.3 using 4-aminophenylboronic acid (**5c**) (0.090 mg, 0.66 µmol), except the reaction was run at 60 °C for 1 h. Analysis by radio-HPLC using method D gave a radiochemical conversion of 74%.

2-[^{125}I]Iodotoluene (6d)

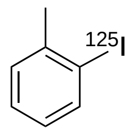

The reaction was carried out according to the general procedure as described in section 3.3 using 2-methylphenylboronic acid (**5d**) (0.090 mg, 0.66 μmol). The reaction was run at room temperature for 10 minutes. Analysis by radio-HPLC using method A gave a radiochemical conversion of 100%.

4-[^{125}I]Iodobiphenyl (6e)

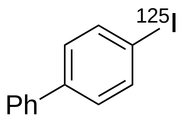

The reaction was carried out according to the general procedure as described in section 3.3 using 4-biphenylboronic acid (**5e**) (0.13 mg, 0.66 μmol). The reaction was run at room temperature for 10 minutes. Analysis by radio-HPLC using method A gave a radiochemical conversion of 96%.

2-[^{125}I]Iodonaphthalene (6f)

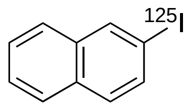

The reaction was carried out according to the general procedure as described in section 3.3 using 2-naphthylboronic acid (**5f**) (0.11 mg, 0.66 μmol). The reaction was run at room temperature for 10 minutes. Analysis by radio-HPLC using method A gave a radiochemical conversion of 99%.

[^{125}I]Iodo-3-nitrobenzene (6g)

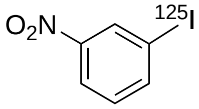

The reaction was carried out according to the general procedure as described in section 3.3 using 3-nitrophenylboronic acid (**5g**) (0.11 mg, 0.66 μmol). The reaction was run at room temperature for 10 minutes. Analysis by radio-HPLC using method A gave a radiochemical conversion of 100%.

4-[^{125}I]Iodobenzonitrile (6h)

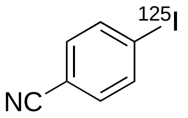

The reaction was carried out according to the general procedure as described in section 3.3 using 4-cyanophenylboronic acid (**5h**) (0.10 mg, 0.66 μmol). The reaction was run at room temperature for 10 minutes. Analysis by radio-HPLC using method A gave a radiochemical conversion of 100%.

4-[^{125}I]Iodobenzaldehyde (6i)

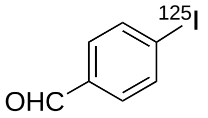

The reaction was carried out according to the general procedure as described in section 3.3 using 4-formylphenylboronic acid (**5i**) (0.10 mg, 0.66 μmol). The reaction was run at room temperature for 10 minutes. Analysis by radio-HPLC using method A gave a radiochemical conversion of 98%.

Ethyl 4-[^{125}I]iodobenzoate (6j)

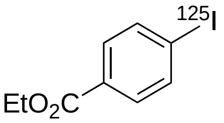

The reaction was carried out according to the general procedure as described in section 3.3 using 4-ethoxycarbonylphenylboronic acid (**5j**) (0.13 mg, 0.66 μmol). The reaction was run at room temperature for 10 minutes. Analysis by radio-HPLC using method A gave a radiochemical conversion of 100%.

3-[^{125}I]Iodo-4-methoxybenzoic acid (6k)

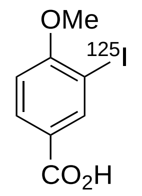

The reaction was carried out according to the general procedure as described in section 3.3 using 2-methoxy-5-carboxyphenylboronic acid (**5k**) (0.13 mg, 0.66 μmol), except the reaction was run at room temperature for 2 h. Analysis by radio-HPLC using method A gave a radiochemical conversion of 74%.

1-Bromo-4-[^{125}I]iodobenzene (6l)

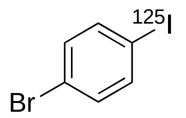

The reaction was carried out according to the general procedure as described in section 3.3 using 4-bromophenylboronic acid (**5l**) (0.13 mg, 0.66 μmol). The reaction was run at room temperature for 10 minutes. Analysis by radio-HPLC using method A gave a radiochemical conversion of 100%.

2-Fluoro-[^{125}I]iodobenzene (6m)

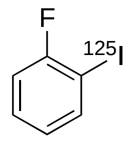

The reaction was carried out according to the general procedure as described in section 3.3 using 2-fluorophenylboronic acid (**5m**) (0.090 mg, 0.66 μmol). The reaction was run at room temperature for 10 minutes. Analysis by radio-HPLC using method A gave a radiochemical conversion of 100%.

(*E*)-2-[^{125}I]Iodoethenylbenzene (6n)

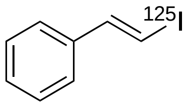

The reaction was carried out according to the general procedure as described in section 3.3 using *trans*-2-phenylvinylboronic acid (**5n**) (0.10 mg, 0.66 µmol). The reaction was run at room temperature for 10 minutes. Analysis by radio-HPLC using method A gave a radiochemical conversion of 97%.

2-[^{125}I]Iodothiophene (6o)

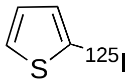

The reaction was carried out according to the general procedure as described in section 3.3 using 2-thienylboronic acid (**5o**) (0.080 mg, 0.66 µmol). The reaction was run at room temperature for 10 minutes. Analysis by radio-HPLC using method A gave a radiochemical conversion of 92%.

4-[^{125}I]Iodopyridine (6p)

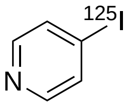

The reaction was carried out according to the general procedure as described in section 3.3 using 4-pyridinylboronic acid (**5p**) (0.08 mg, 0.66 µmol), except with the addition of acetonitrile (100 µL). The reaction was run at room temperature for 10 minutes. Analysis by radio-HPLC using method A and B gave a radiochemical conversion of 97%.

5-[^{125}I]Iodouracil (8)

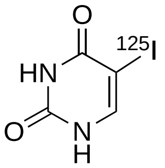

To a vial containing uracil-5-boronic acid (**7**) (0.10 mg, 0.66 µmol) in dimethyl sulfoxide (40 µL) was added [Cu(OAc)(phen)$_2$]OAc (7.2 µg, 0.013 µmol) in methanol:water (4:1, 100 µL) from a stock

solution. The solution was then mixed by vortex for 30 seconds. To the reaction vessel was added [^{125}I]NaI (2.9 MBq, 5 µL of batch 2 [^{125}I]NaI solution). The reaction mixture was mixed by vortex for 5 seconds, heated to 60 °C, and then left for 1.5 h. An aliquot (10 µL) of the reaction mixture was removed and diluted to a total volume of 500 µL using a 1:1 mixture of 0.1% trifluoroacetic acid in water and 0.1% trifluoroacetic acid in acetonitrile. Analysis of the aliquot solution by radio-HPLC using method C gave a radiochemical conversion of 92%.

***N*-(3-[^{125}I]Iodobenzyl)guanidine ([^{125}I]MIBG, 13)**

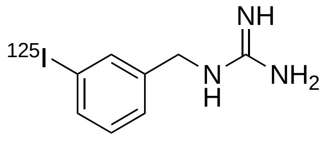

Synthesis of [^{125}I]MIBG (**13**) from di-*N*-boc-protected boronic acid precursor (**11**):

The radioiodination reaction was carried out according to the general procedure as described in section 3.3 using {3-[2,3-bis(*tert*-butoxycarbonyl)guanidino]methylphenyl}boronic acid (**11**) (0.26 mg, 0.66 µmol). The reaction was run at room temperature for 10 minutes. After 10 minutes had elapsed, 6 M aqueous hydrochloric acid (0.11 mL, 0.66 mmol) was added to the reaction mixture. The reaction was mixed by vortex for 5 seconds, heated to 60 °C and then left for 10 minutes. Analysis by radio-HPLC using method D gave a radiochemical conversion of 95%.

Synthesis of [^{125}I]MIBG (**13**) from unprotected boronic acid precursor (**14**):

The reaction was carried out according to the general procedure as described in section 3.3 using [3-(guanidinomethyl)phenyl]boronic acid hydrochloride (**14**) (0.15 mg, 0.66 µmol), except the reaction was run at room temperature for 25 minutes. Analysis by radio-HPLC using method D gave a radiochemical conversion of 96%.

3.5. General Experimental Information for Radioiodination with [^{123}I]NaI

Sodium [^{123}I]iodide was purchased from GE Healthcare (product number 1139121) as no-carrier added [^{123}I]NaI in a 0.05 M aqueous solution of sodium hydroxide with a concentration of 24 GBq/mL. This solution was diluted with methanol:water (4:1) to give a total volume of 100 µL and then 50 µL of this solution was transferred to a second V-vial producing two batches (batches 1a and 1b). Radiochemical conversion and molar activity were determined by semi-preparative radio-HPLC

analysis of the crude reaction mixture. Radiochemical purity was determined by radio-HPLC analysis of the formulated product (see Section 3.2. for equipment details).

3.6. Semi-preparative Radio-HPLC Method for Determination of Radiochemical Yield, Purification of [^{123}I]MIBG (1), and Determination of Molar Activity

Semi-preparative HPLC was performed with a Dionex Ultimate 3000 HPLC system equipped with a Knauer Advanced Scientific Instruments Smartline UV Detector 2500 and a photomultiplier tube (PMT) connected to a Lab Logic Flow-Count radiodetector, using a Jones Genesis 4 µm C18 column (150 × 10 mm) with a 10 mm guard cartridge, UV 254 nm and flow 3 mL/min. The mobile phase for the elution of [^{123}I]MIBG (**1**) was 0.1% trifluoroacetic acid in water and 0.1% trifluoroacetic acid in acetonitrile, with a gradient profile as shown below.

Time (mins)	%MeCN (0.1% TFA)	%H_2O (0.1% TFA)
**0–20**	25	75
**20.0–20.1**	25–95	75–5
**20.1–35**	95	5
**35–35.1**	95–25	5–75
**35.1–40**	25	75

3.7. Determination of Molar Activity of [^{123}I]MIBG (1)

For each synthesis of *N*-(3-[^{123}I]iodobenzyl)guanidine ([^{123}I]MIBG, **1**), the quantity of [^{123}I]MIBG (**1**) produced was below the UV limit of detection. Therefore, the molar activity of the product could not be accurately determined using a calibration curve obtained from HPLC analysis of *N*-(3-[^{127}I]iodobenzyl)guanidine. Instead, using the equation shown below, the UV limit of detection for *N*-(3-[^{127}I]iodobenzyl)guanidine was calculated to be 5.43136 × 10^{-3} µmol and this value was used to determine the molar activity of [^{123}I]MIBG (**1**) synthesized.

$$Limit\ of\ Detection = 3 \times \left[\frac{\left(\frac{Baseline\ Noise\ (mV)}{2} \right) \times\ Moles\ of\ Sample\ (\mu mol)}{Peak\ Height\ (mV)} \right]$$

3.8. Experimental Procedure for the Synthesis and Purification of [^{123}I]MIBG (1)

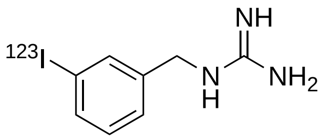

To a vial containing [3-(guanidinomethyl)phenyl]boronic acid hydrochloride (**14**) (0.15 mg, 0.66 µmol) was added [Cu(OAc)(phen)$_2$]OAc (7.2 µg, 0.013 µmol) in methanol:water (4:1, 50 µL) from a stock solution. The solution was mixed by vortex for 30 seconds and then transferred to a v-vial containing [^{123}I]NaI in a 0.05 M aqueous solution of sodium hydroxide (27.6–28.9 MBq, 50 µL). The reaction mixture was mixed by vortex for 3 seconds, heated to 25 °C, and then left for 25 minutes. The crude mixture was removed from the v-vial by syringe and diluted with 25% acetonitrile in water (0.1% trifluoroacetic acid) (200 µL). The crude product was purified by semi-preparative HPLC. The fraction containing *N*-(3-[^{123}I]iodobenzyl)guanidine (**1**) was evaporated to dryness, and then reconstituted in 0.9% saline (5 mL) to afford *N*-(3-[^{123}I]iodobenzyl)guanidine (**1**) in 74 ± 2% radiochemical yield (determined using the starting activity of [^{123}I]NaI and measured activity of the isolated product, 19.4 MBq, decayed to the same point in time), with a molar activity of ≥3.74 ± 0.12 GBq/µmol (n = 2) from a total preparation time of 77 minutes. The radiochemical purity of the isolated product was determined by analytical HPLC (method D) and was 100% (n = 2). Co-elution with the UV signal from *N*-(3-[^{127}I]iodobenzyl)guanidine was used to confirm the identity of *N*-(3-[^{123}I]iodobenzyl)guanidine (**1**).

4. Radio-HPLC and UV-Vis HPLC Chromatograms

Analytical HPLC spectra: For each substrate, the radio-trace of the crude reaction mixture (shown in black) is overlaid with the UV-trace of authentic reference compound analyzed separately (shown in blue, y-axis in mAU). The small difference in time of elution is attributed to the time required for sample to flow from the UV detector to the radio-detector.

4-[^{125}I]Iodoanisole (6a)

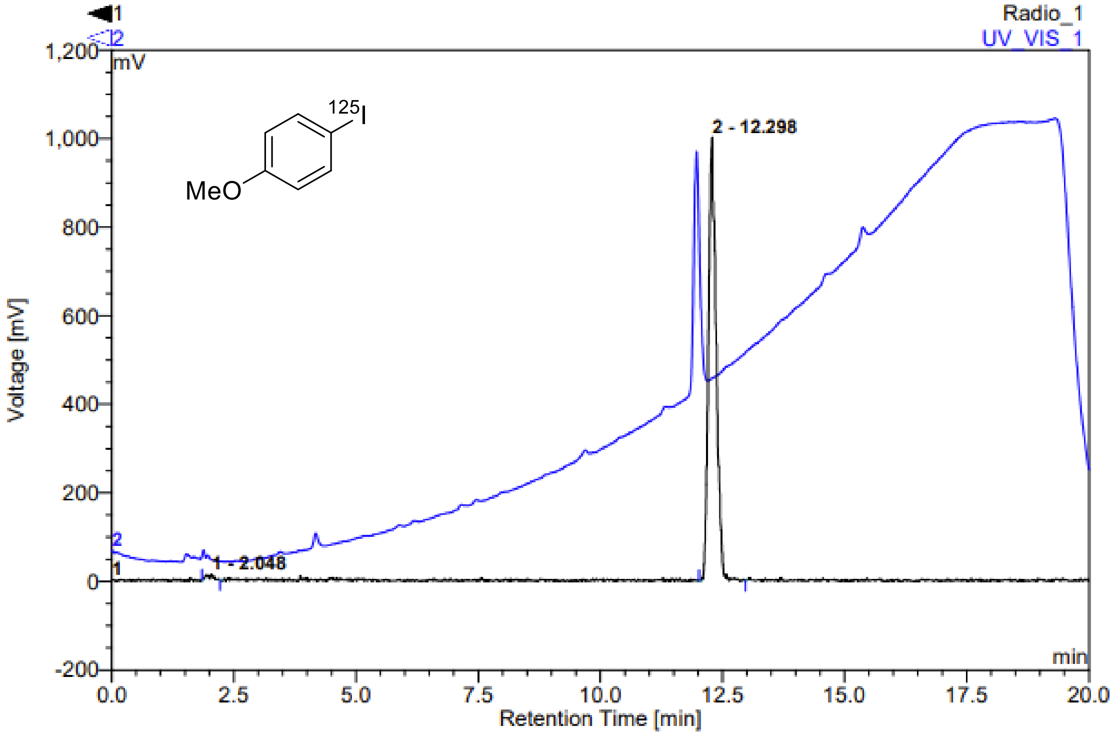

4-[^{125}I]Iodophenol (6b)

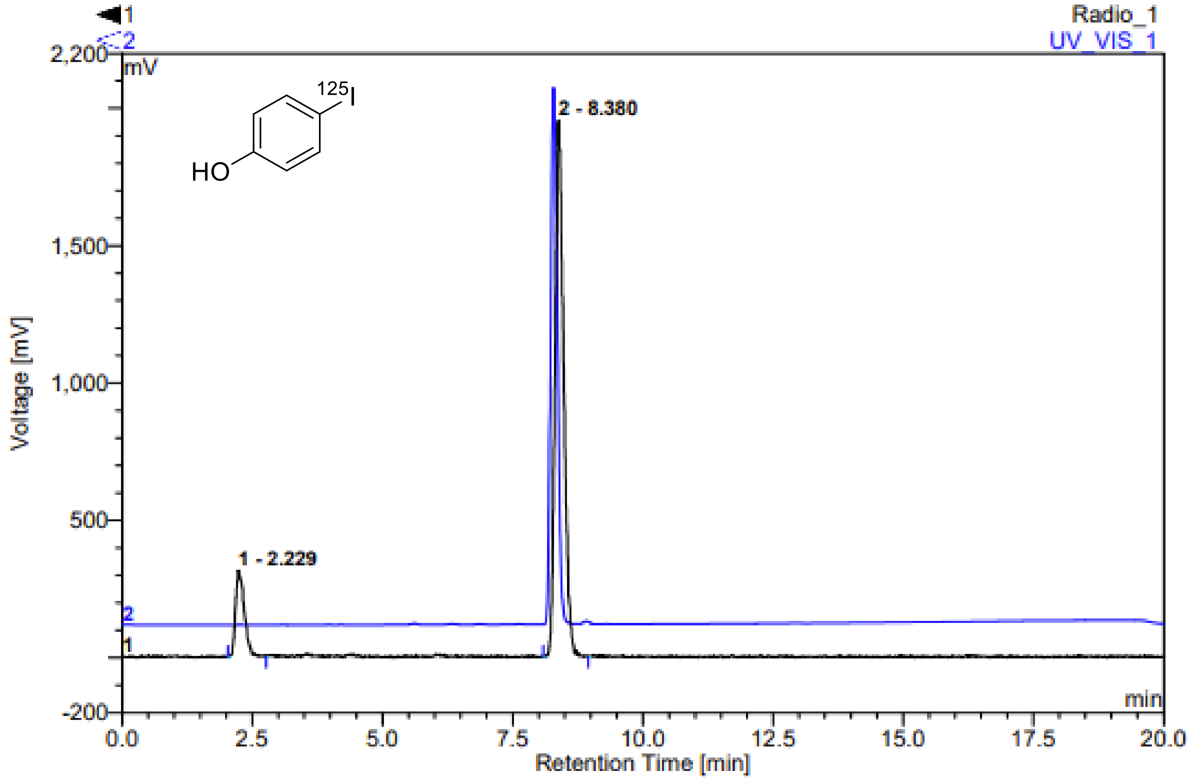

4-[^{125}I]Iodoaniline (6c)

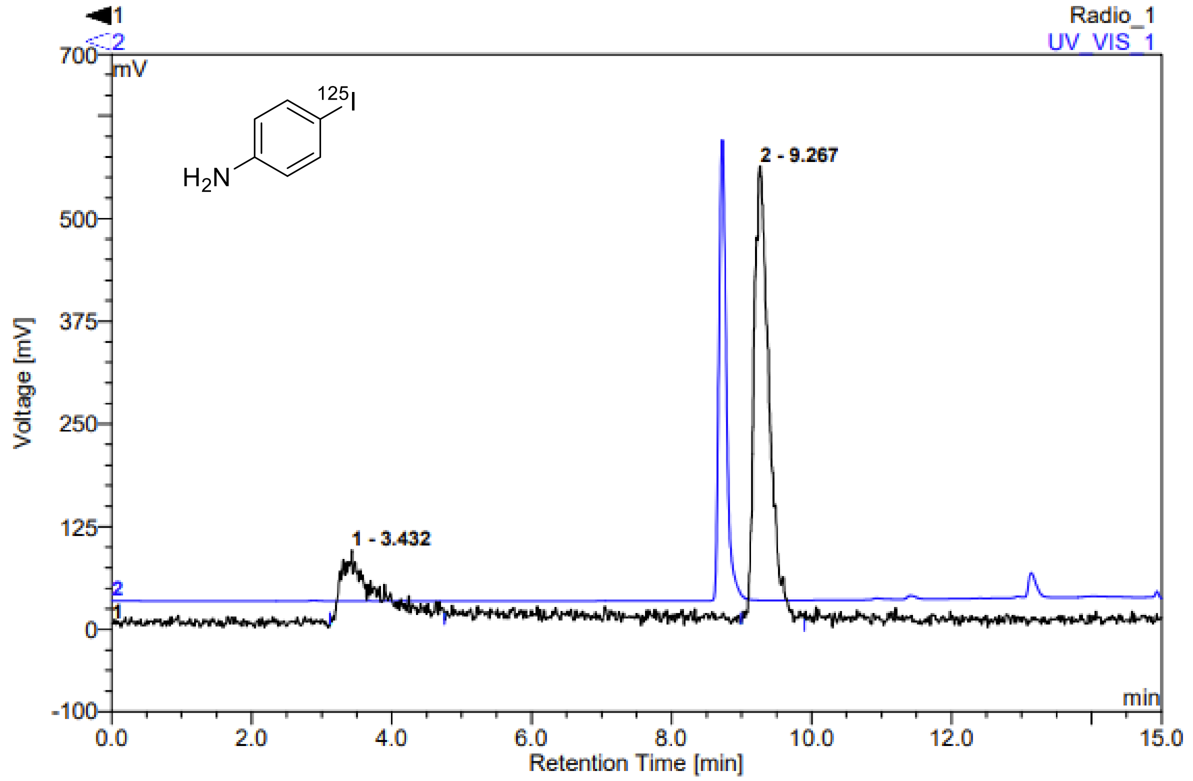

2-[^{125}I]Iodotoluene (6d)

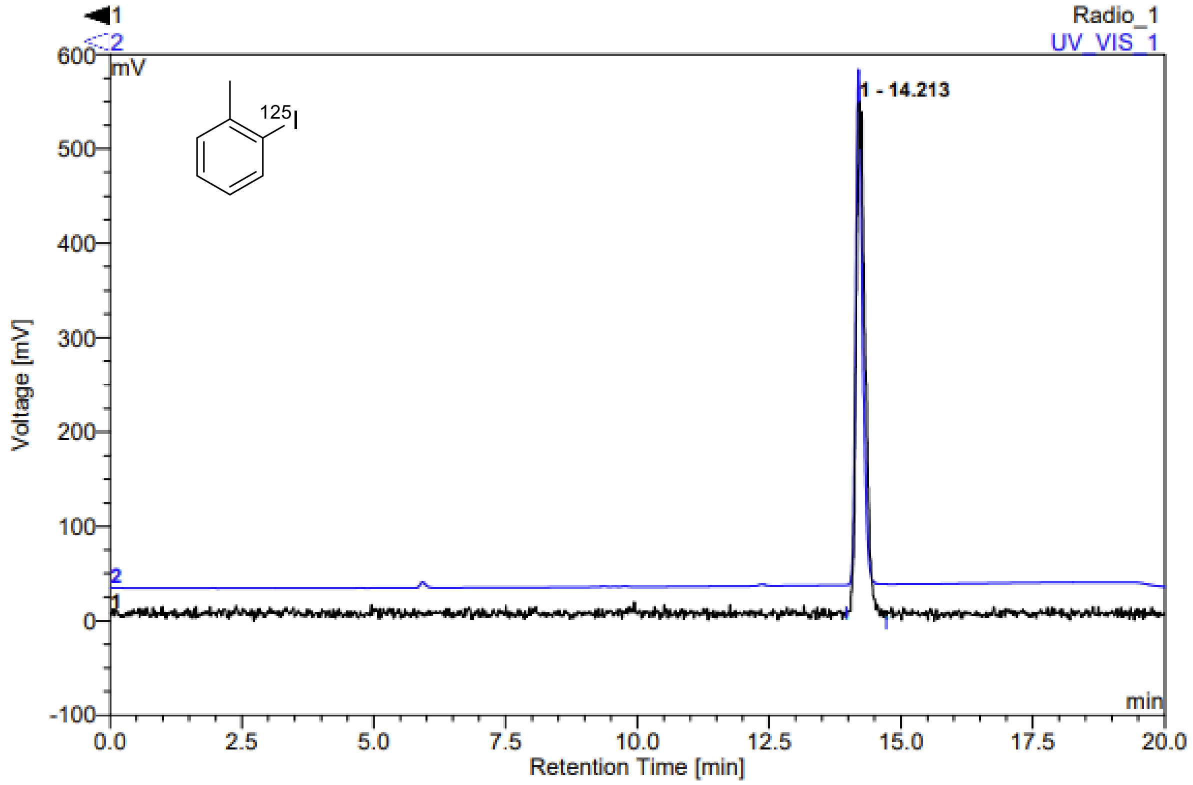

4-[^{125}I]Iodobiphenyl (6e)

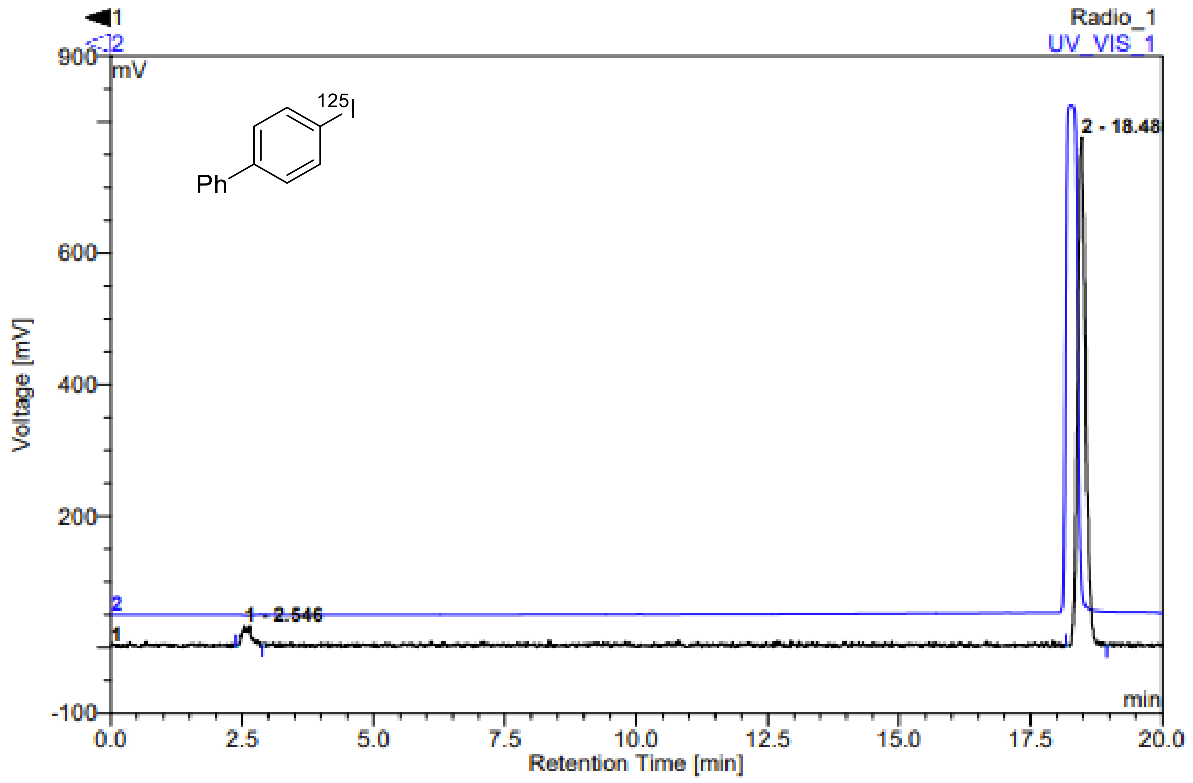

2-[^{125}I]Iodonaphthalene (6f)

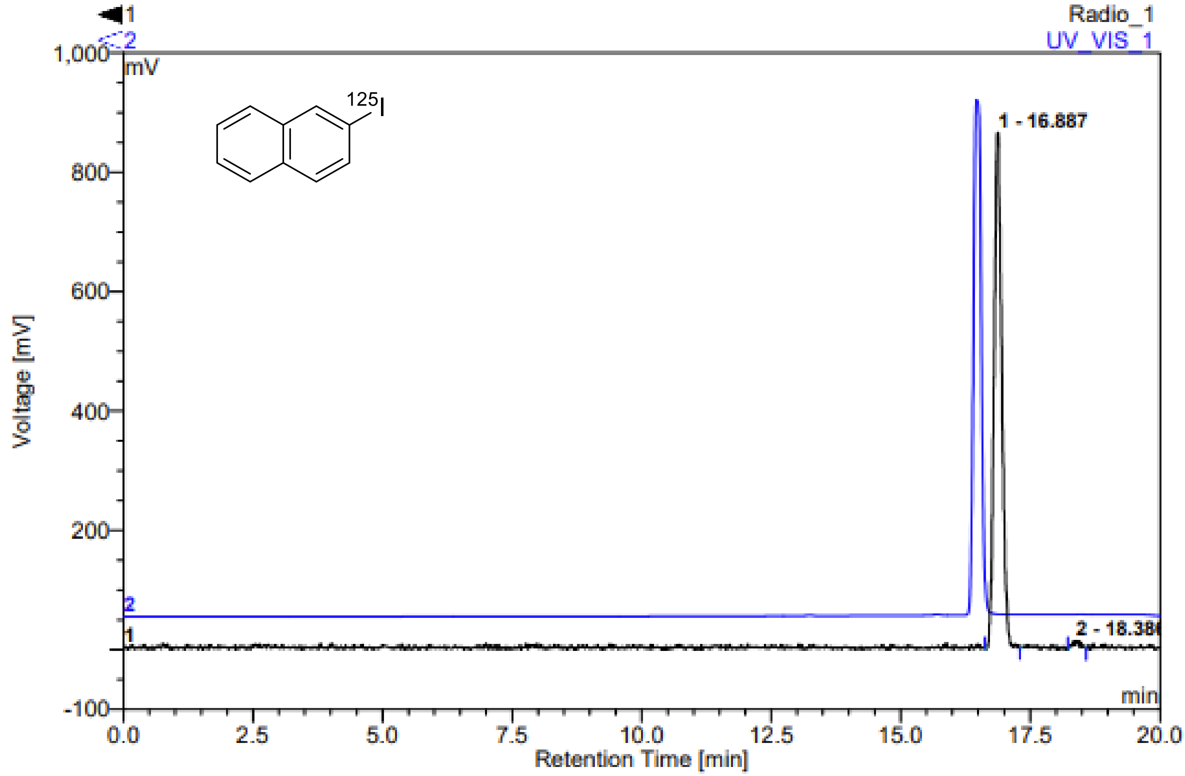

[^{125}I]Iodo-3-nitrobenzene (6g)

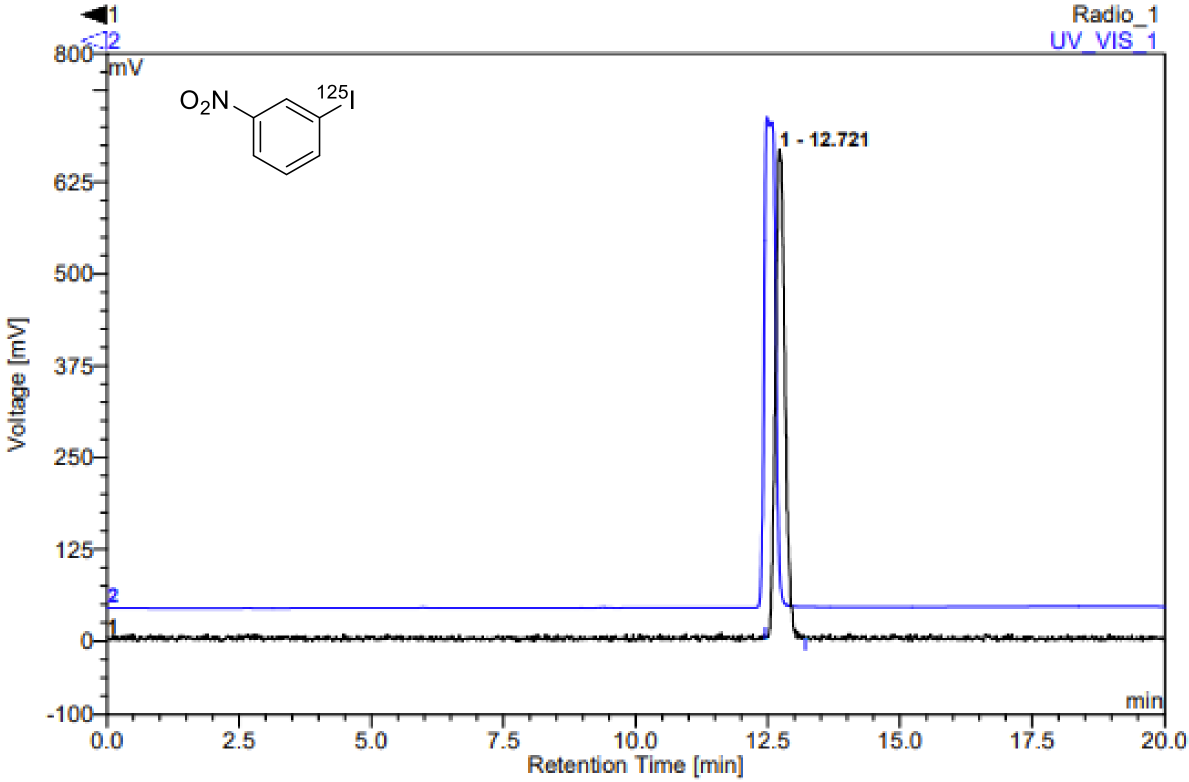

4-[^{125}I]Iodobenzonitrile (6h)

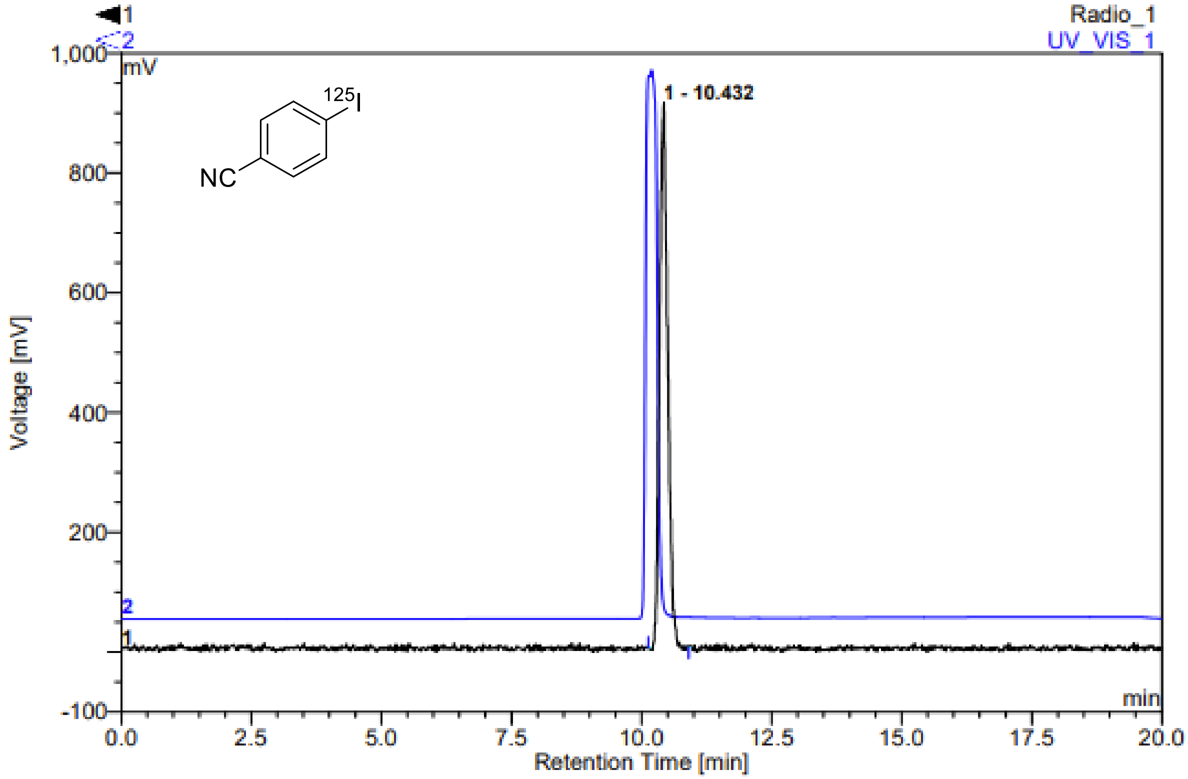

4-[^{125}I]Iodobenzaldehyde (6i)

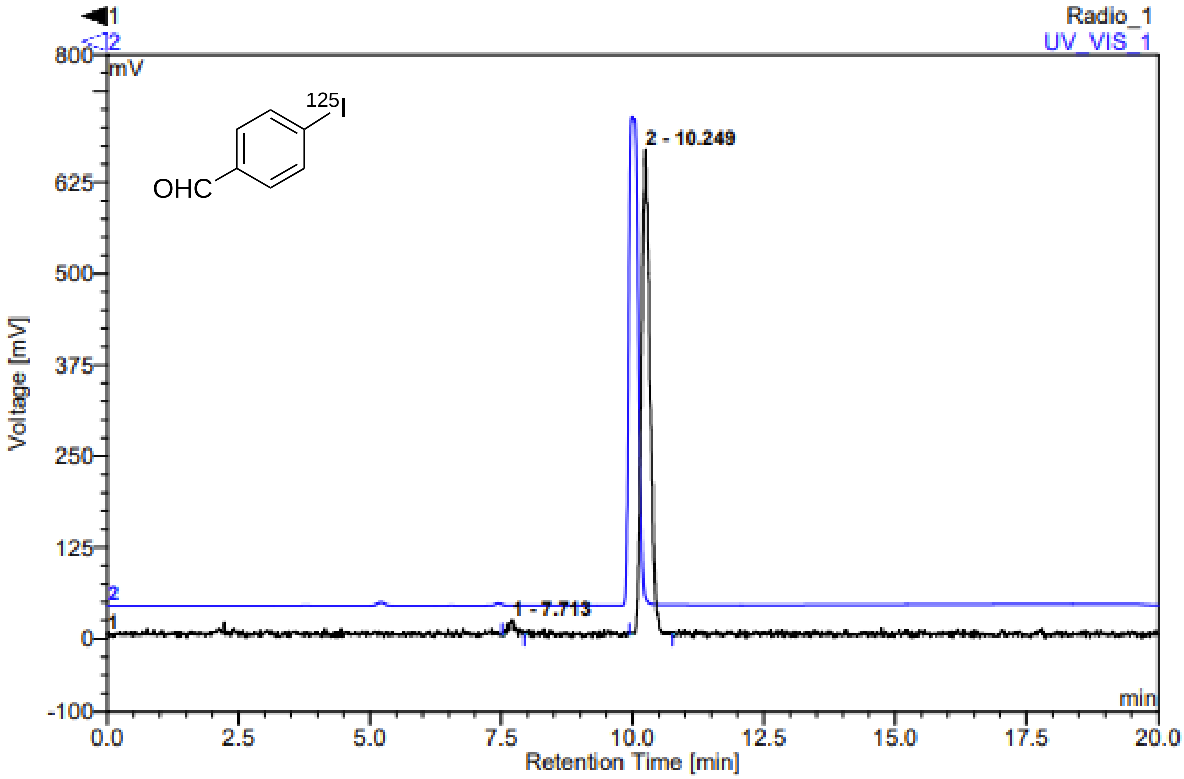

Ethyl 4-[^{125}I]iodobenzoate (6j)

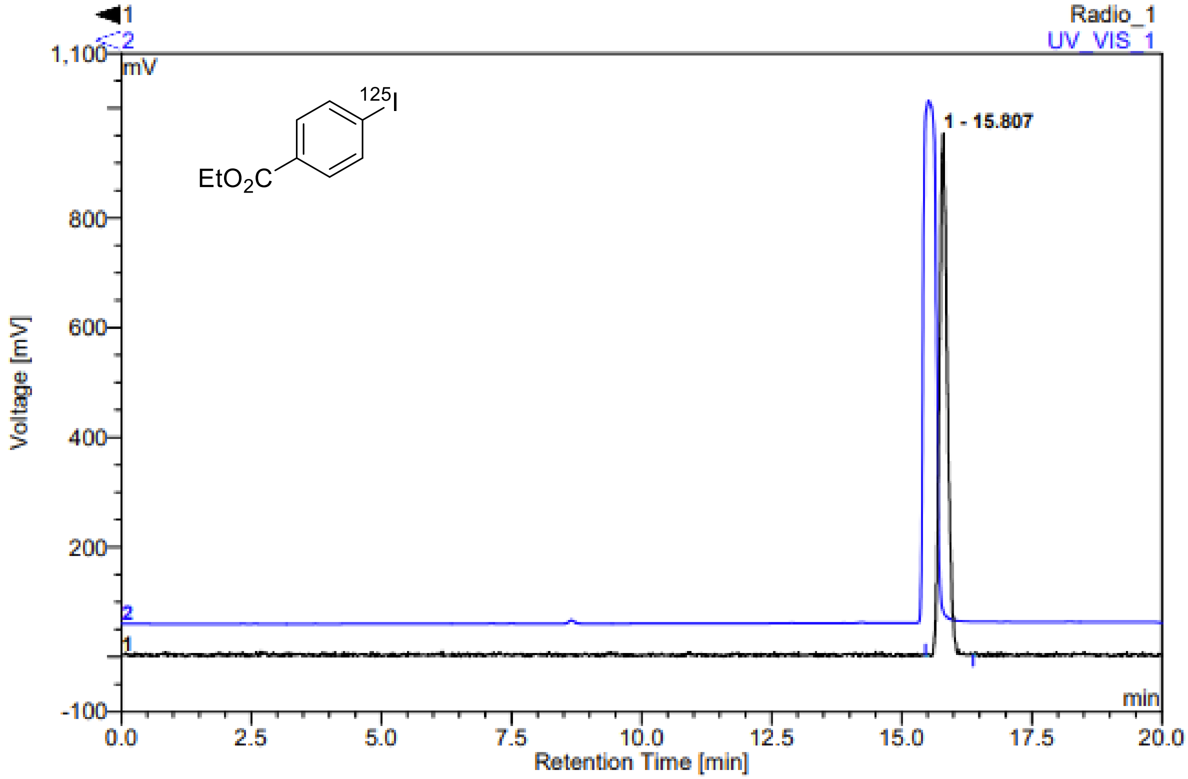

3-[^{125}I]Iodo-4-methoxybenzoic acid (6k)

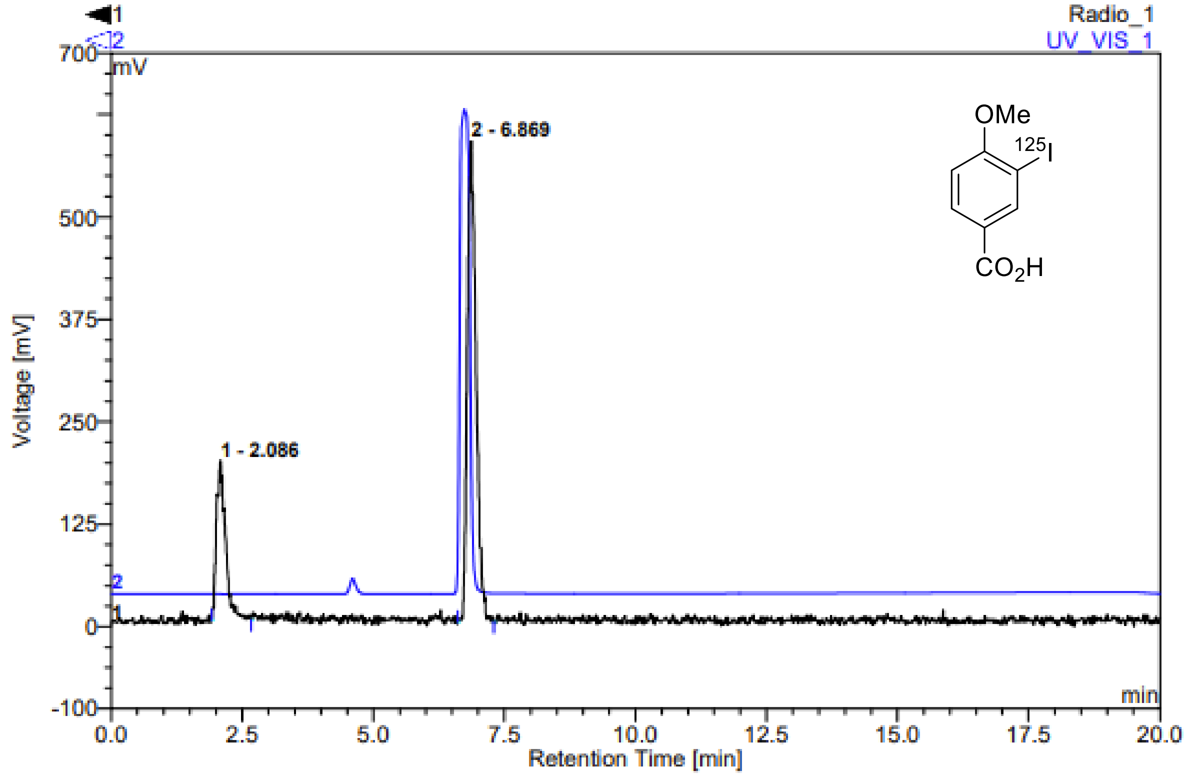

1-Bromo-4-[^{125}I]iodobenzene (6l)

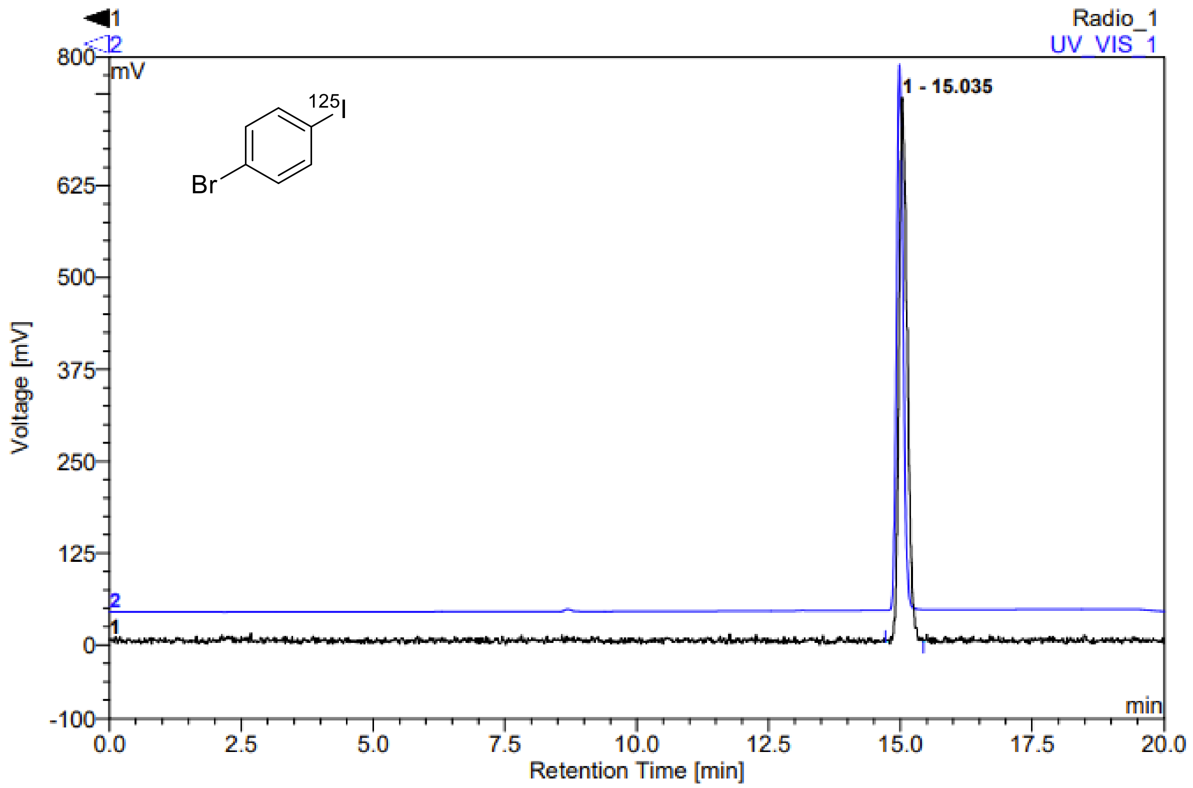

2-Fluoro-[^{125}I]iodobenzene (6m)

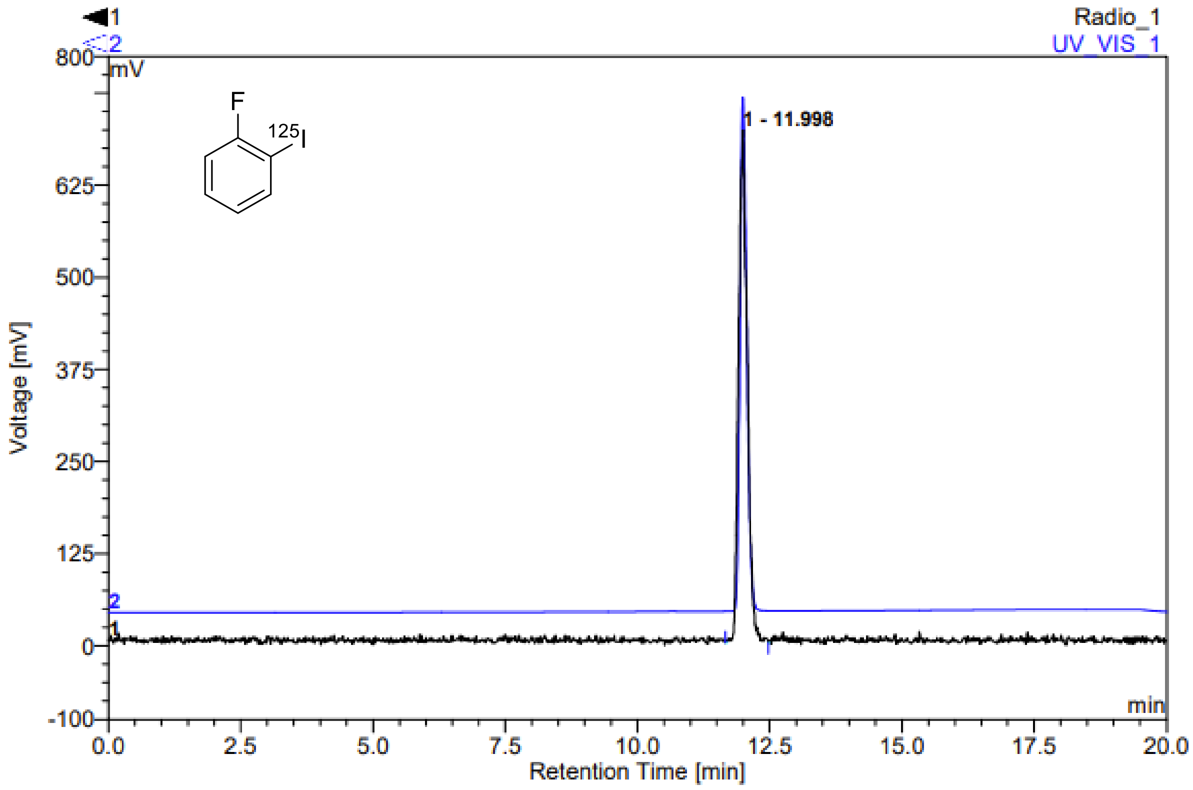

(*E*)-2-[^{125}I]Iodoethenylbenzene (6n)

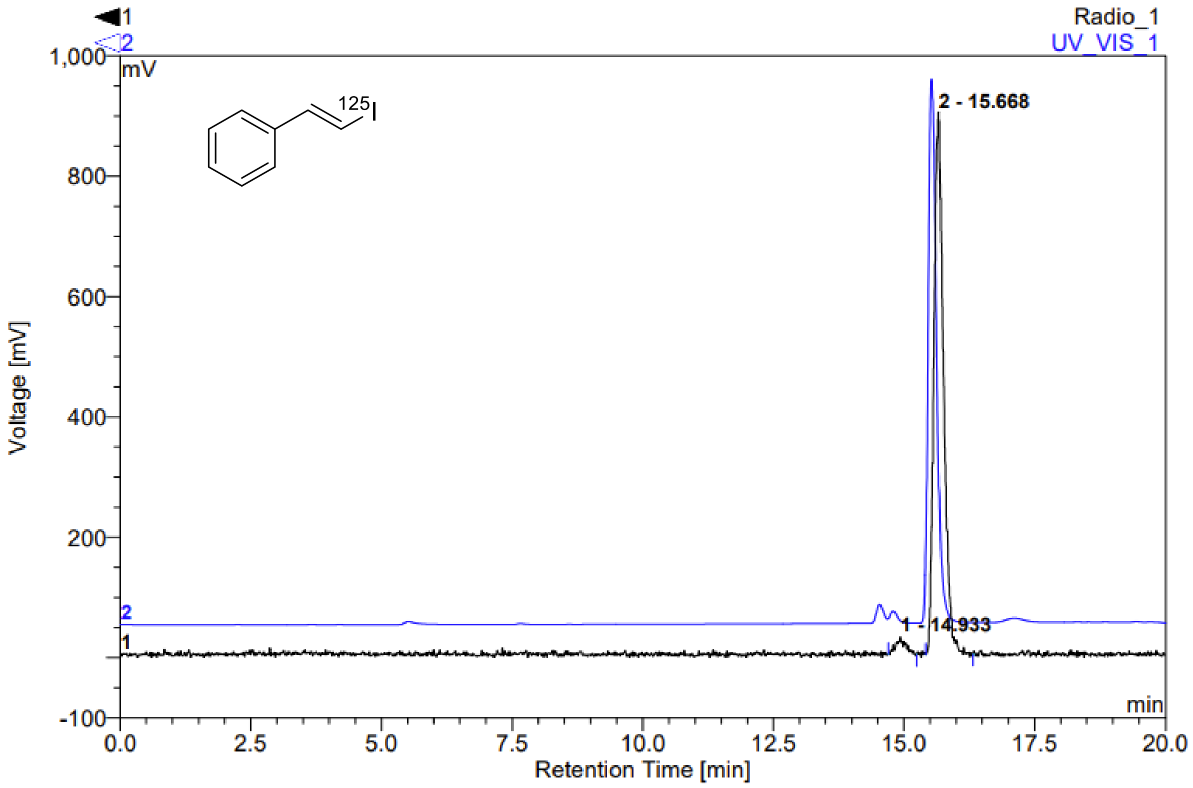

2-[^{125}I]Iodothiophene (6o)

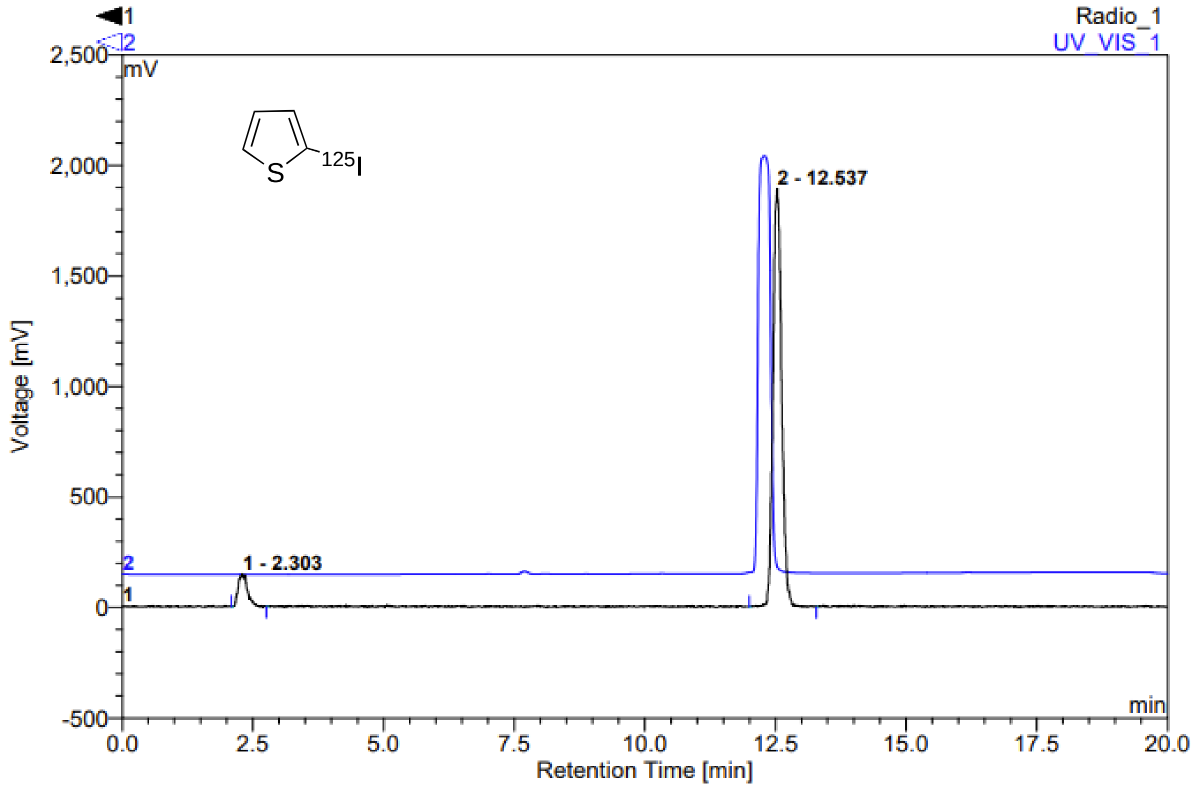

4-[^{125}I]Iodopyridine (6p)

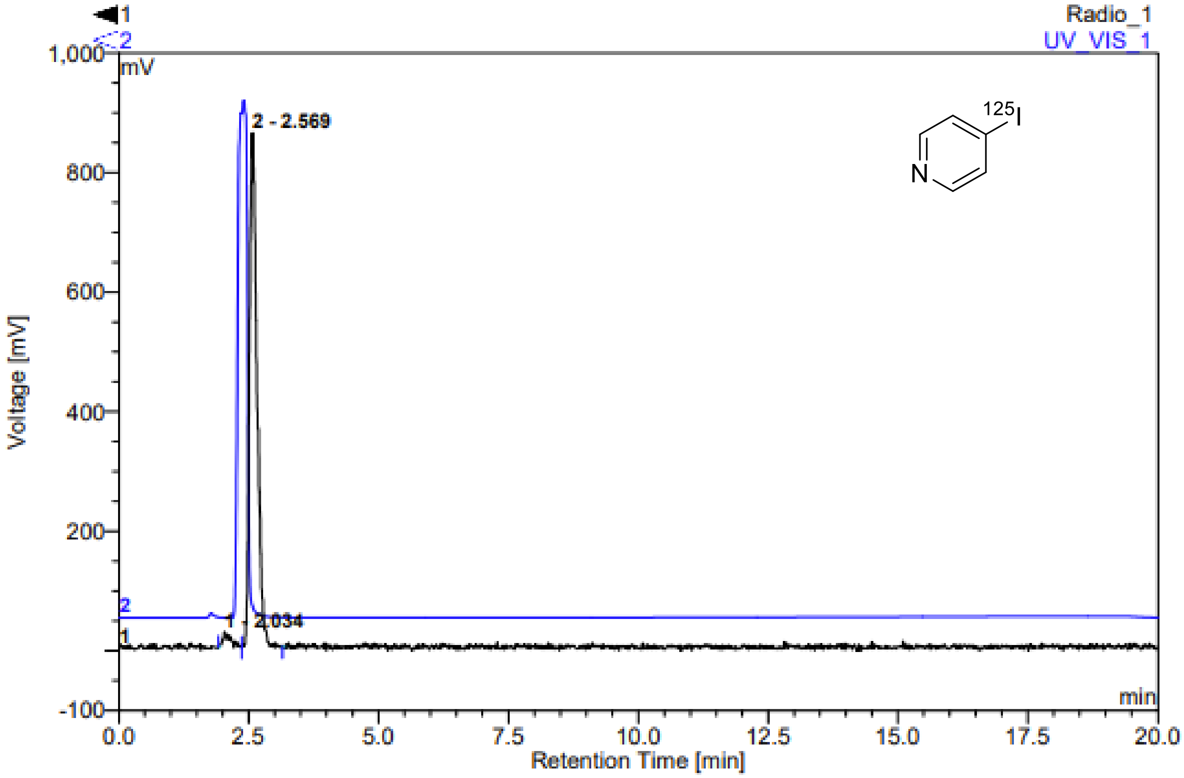

5-[^{125}I]Iodouracil (6q)

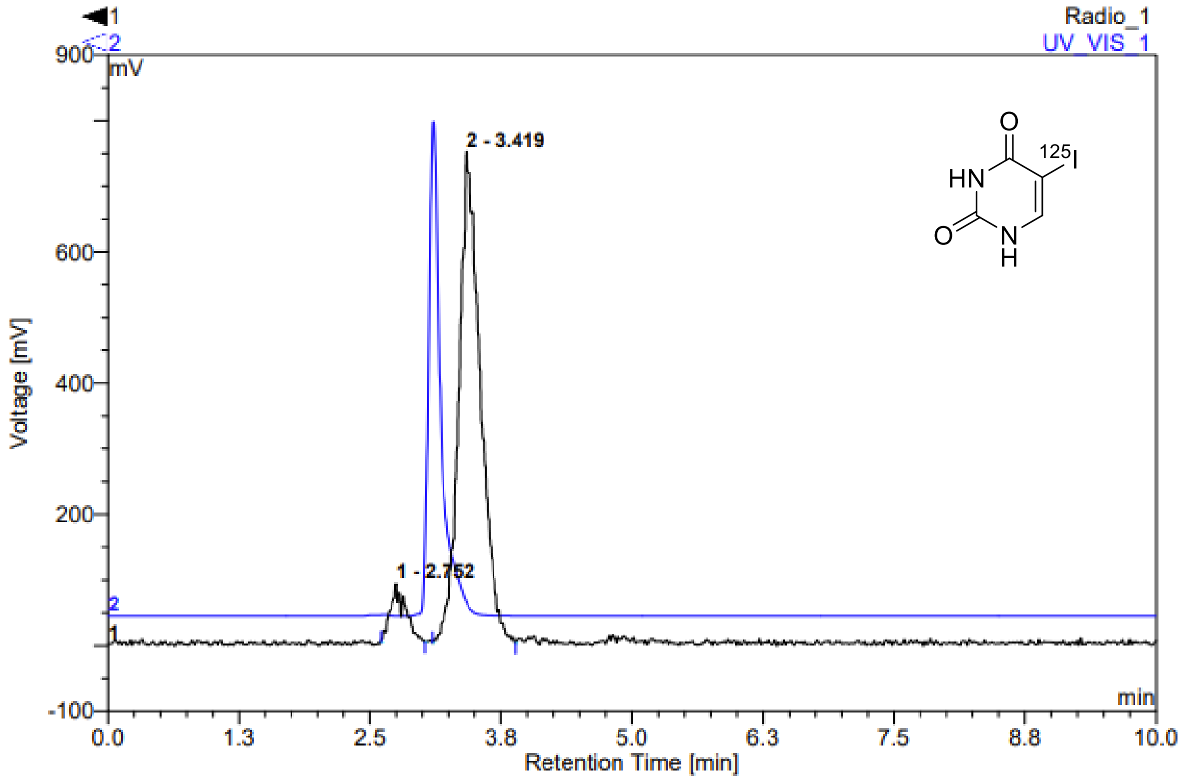

N-(3-[^{125}I]Iodobenzyl)guanidine ([^{125}I]MIBG, 13 from protected precursor, 11)

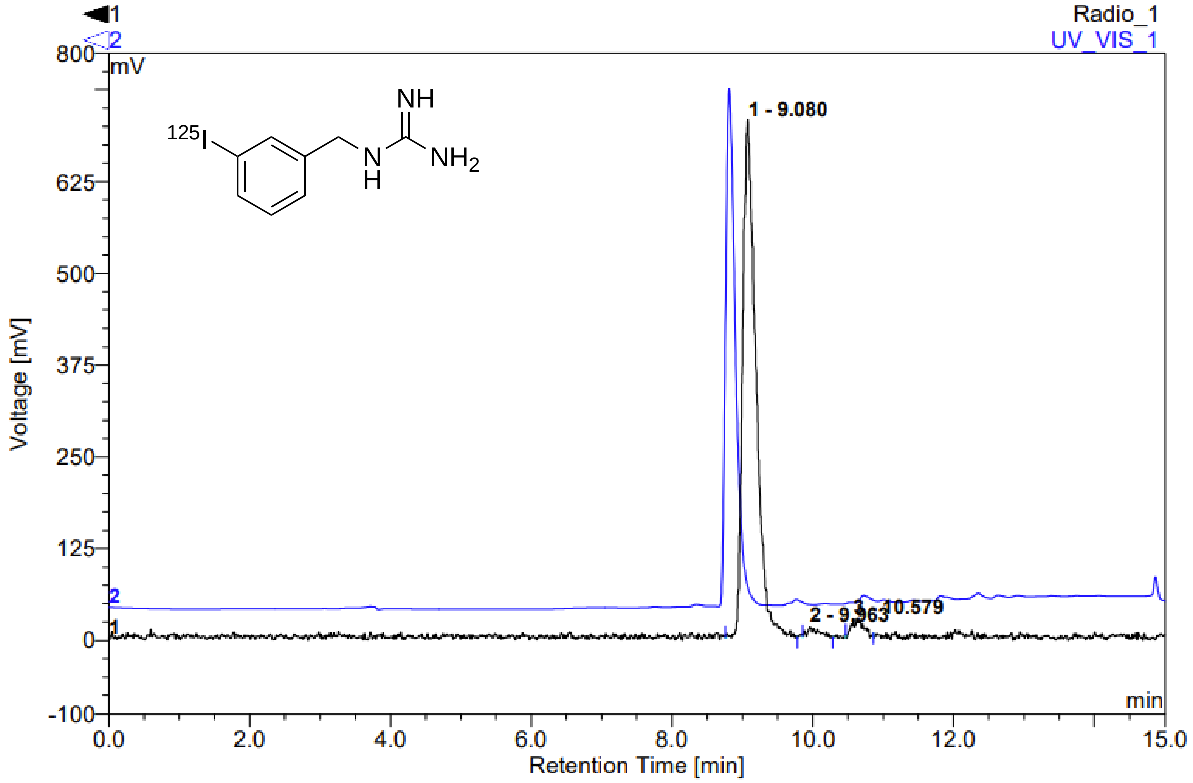

N-(3-[^{125}I]Iodobenzyl)guanidine ([^{125}I]MIBG, 13 from unprotected precursor, 14)

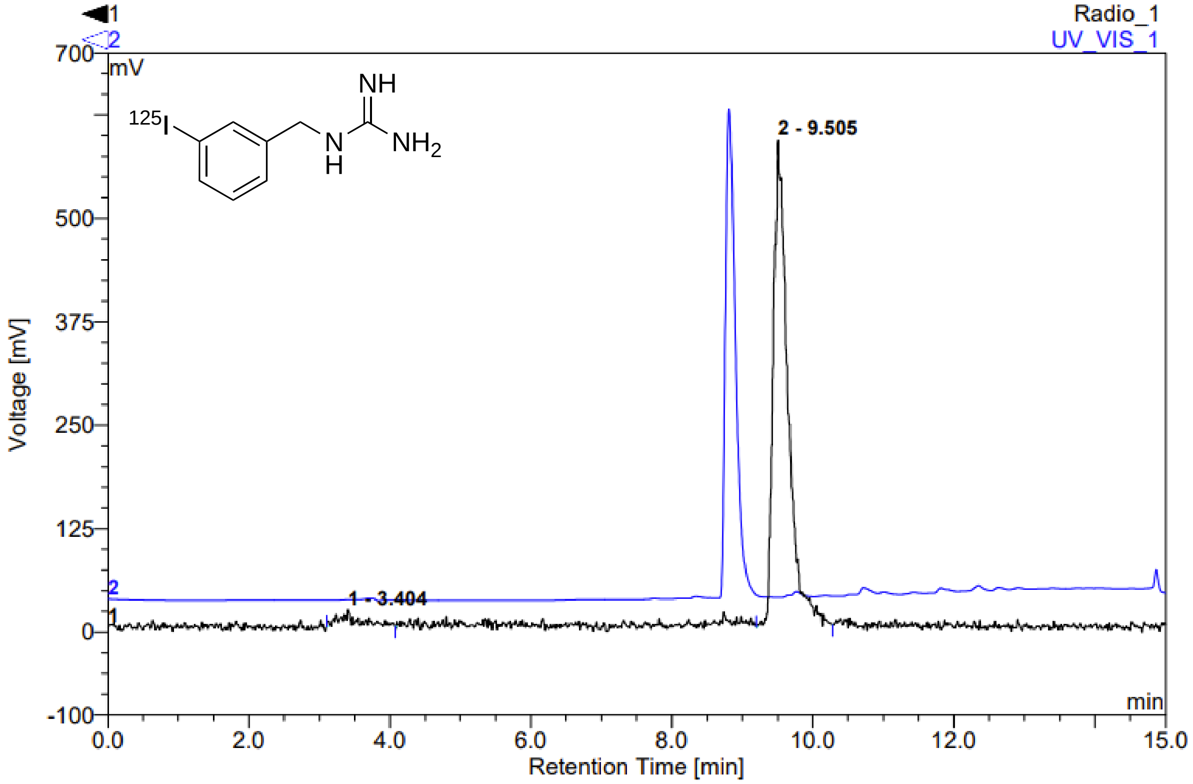

N-(3-[^{123}I]Iodobenzyl)guanidine ([^{123}I]MIBG, 1)

Semi-preparative HPLC spectra: The radio-trace (shown in black) and UV-trace (shown in blue, y-axis in mAU) of the crude reaction mixture.

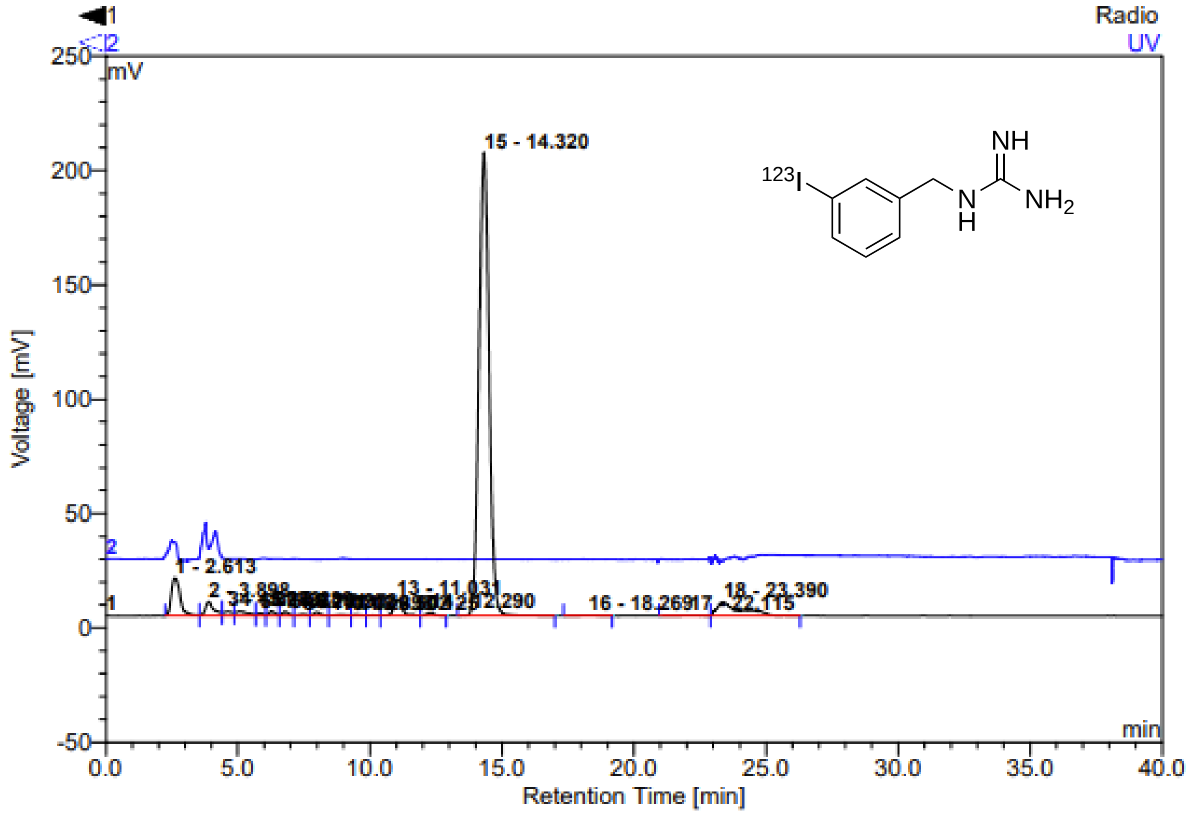

Analytical HPLC spectra of [^{123}I]MIBG (1) after purification and formulation: The radio-trace of the formulated product (shown in black) is overlaid with the UV-trace of authentic reference compound analyzed separately (shown in blue, y-axis in mAU).

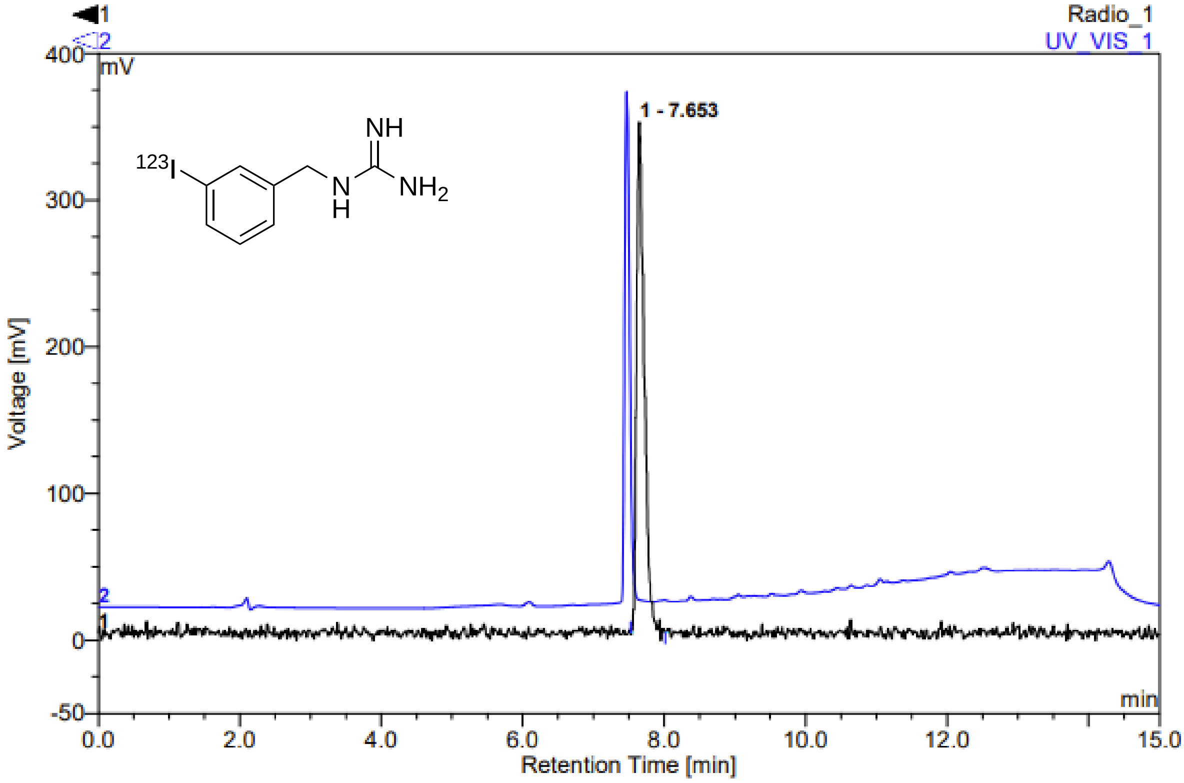

5. References

1. Boehm, P.; Denton, E. H.; Wick, J.; Morandi, B. Intermolecular Pauson-Khand-Type Reaction of Vinyl Iodides with Alkynes and a CO Surrogate. *J. Org. Chem.* **2023**, *88*, 5069–5077.

2. Zhang, P.; Zhuang, R.; Guo, Z.; Su, X.; Chen, X.; Zhang, X. A Highly Efficient Copper-Mediated Radioiodination Approach Using Aryl Boronic Acids. *Chem. Eur. J.* **2016**, *22*, 16783–16786.

3. Wilson, T. C.; McSweeney, G.; Preshlock, S.; Verhoog, S.; Tredwell, M.; Cailly, T.; Gouverneur, V. Radiosynthesis of SPECT Tracers via a Copper Mediated ^{123}I Iodination of (Hetero)Aryl Boron Reagents. *Chem. Commun.* **2016**, *52*, 13277–13280.

4. Jing, B.; Li, L.; Dong, J.; Xu, T. (Acetato-κO)bis(1,10-phenathroline-$\kappa^2 N,N'$)Copper(II) Acetate Heptahydrate. *Acta Cryst*. **2011**, *E67*, m464.

6. ^{1}H and ^{13}C NMR Spectra for all Novel Compounds

^{1}H NMR (400 MHz, CD_3OD)

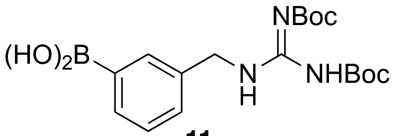

11

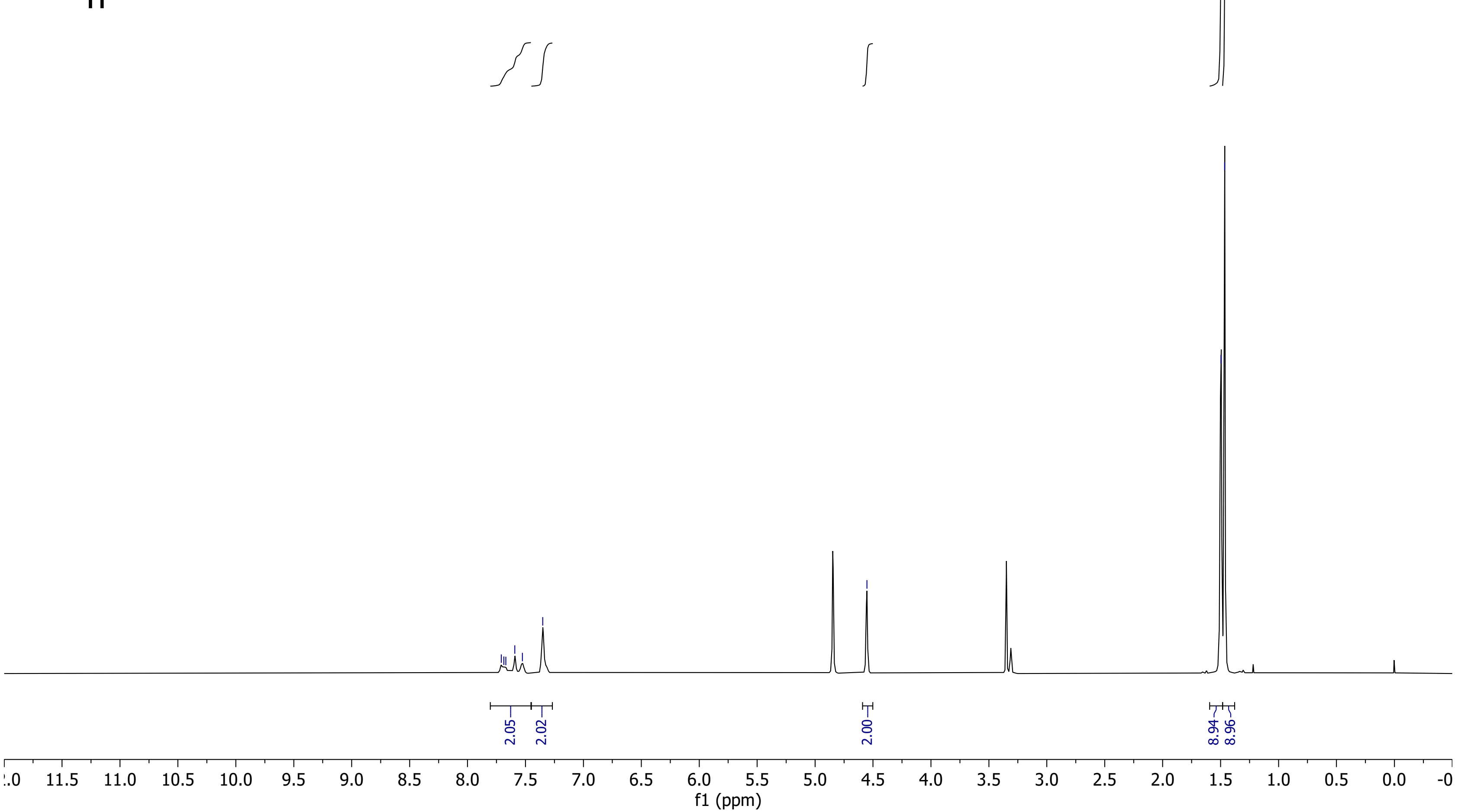

$^{13}C\{^{1}H\}$ NMR (101 MHz, CD_3OD)

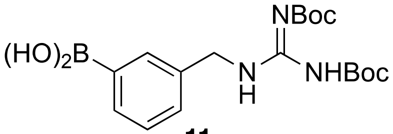

11

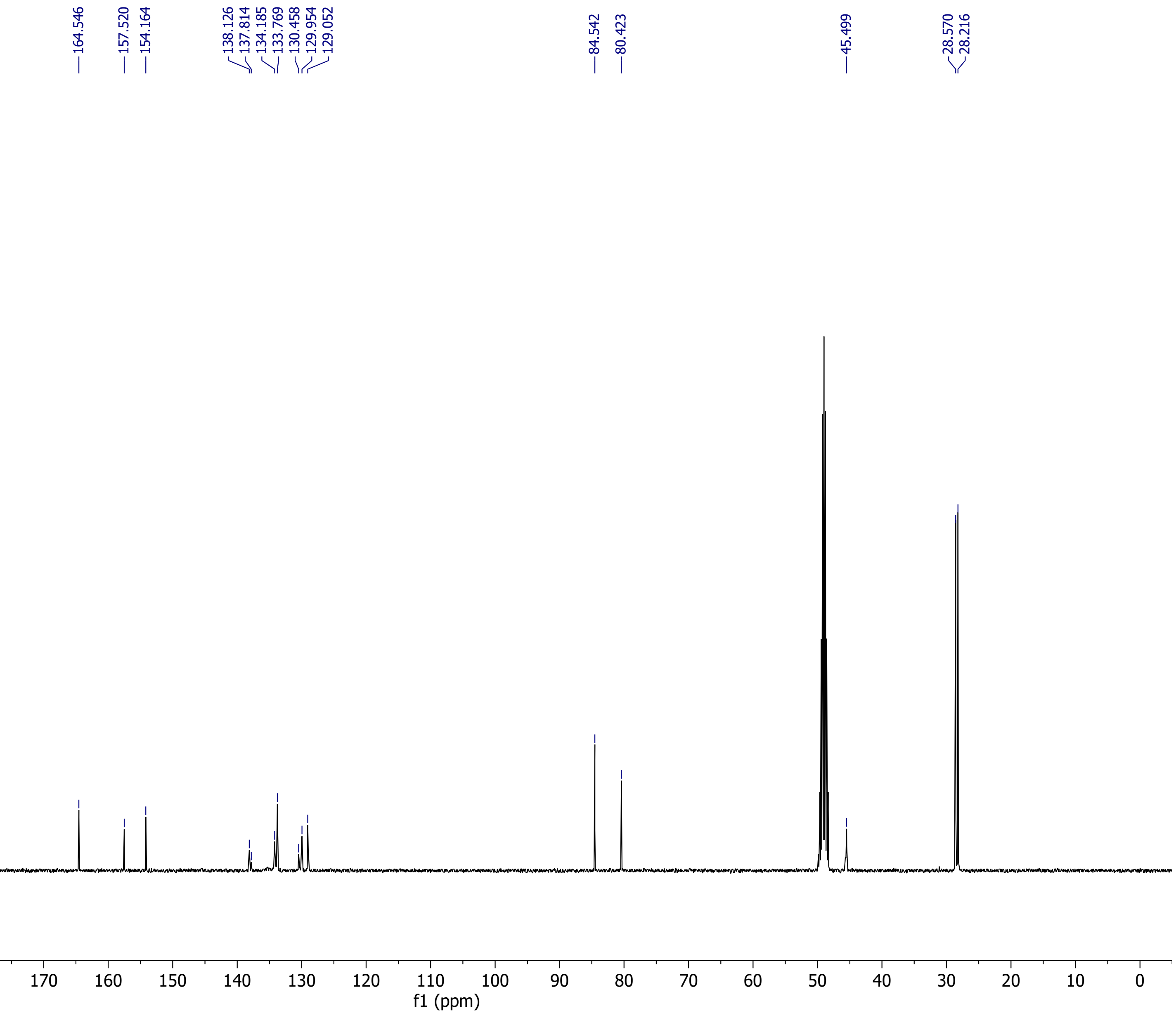

^{1}H NMR (400 MHz, DMSO-d_6)

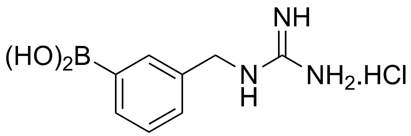

14

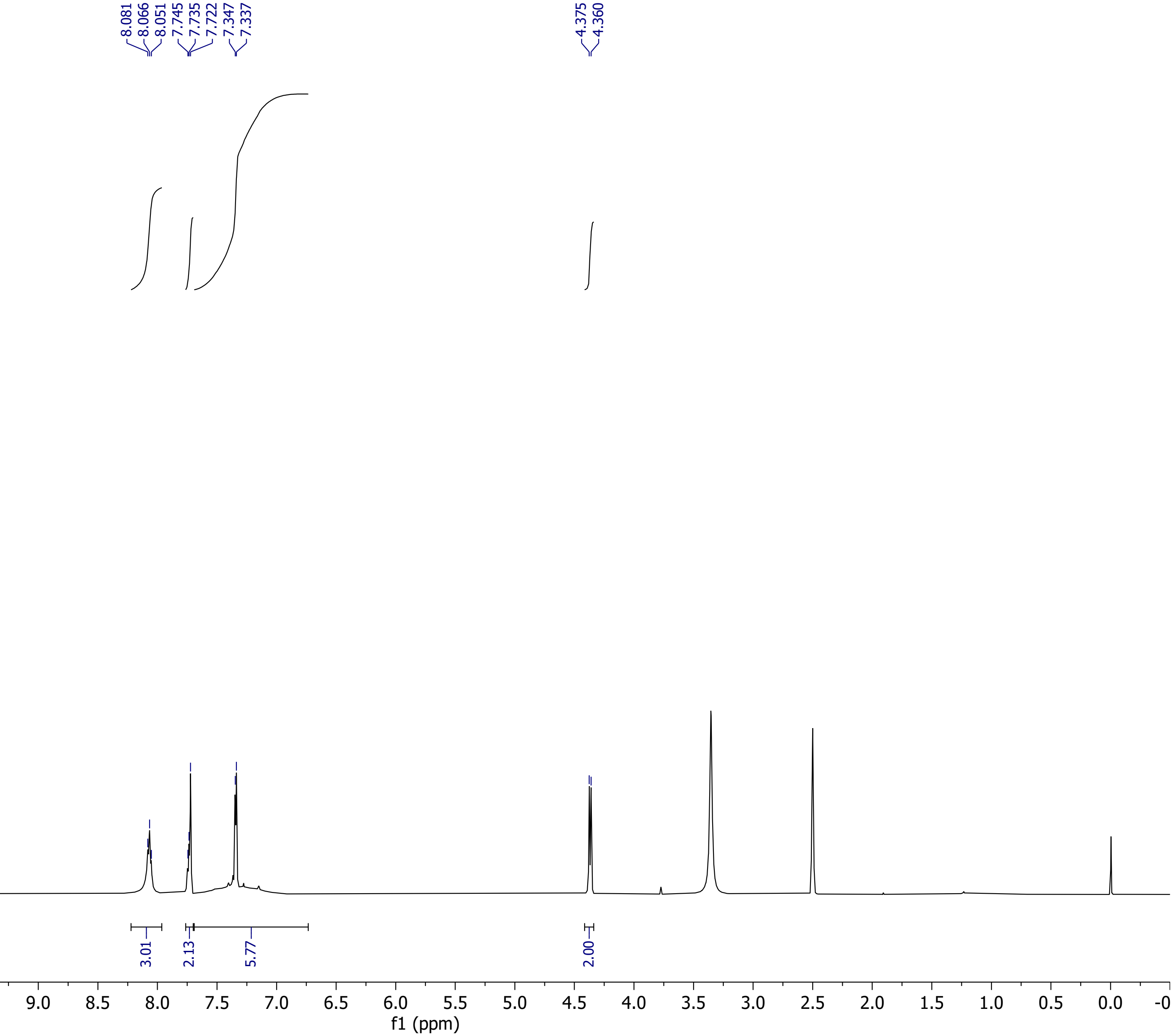

$^{13}C\{^1H\}$ NMR (101 MHz, DMSO-d_6)

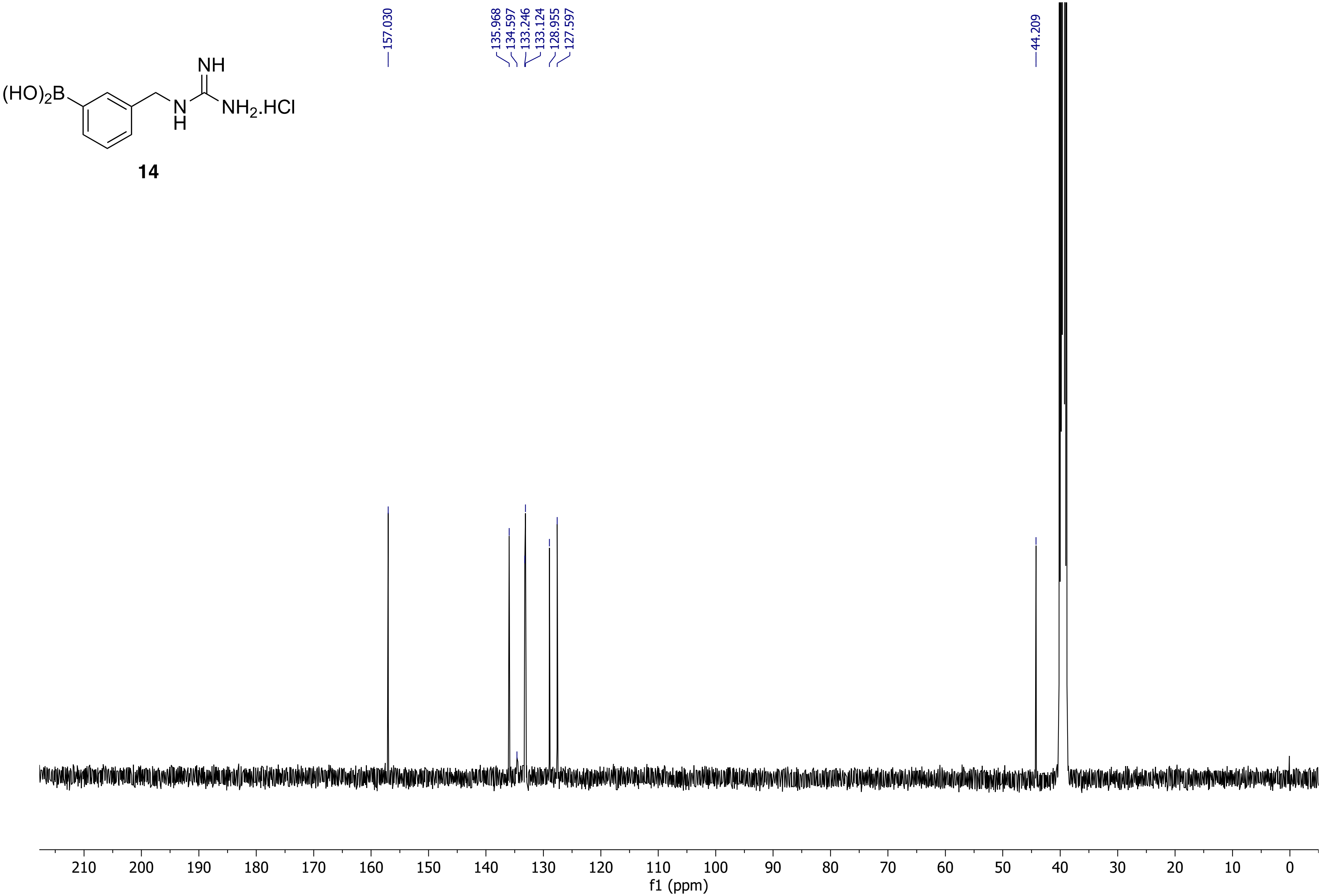

14

^{1}H NMR (400 MHz, CDCl$_3$)

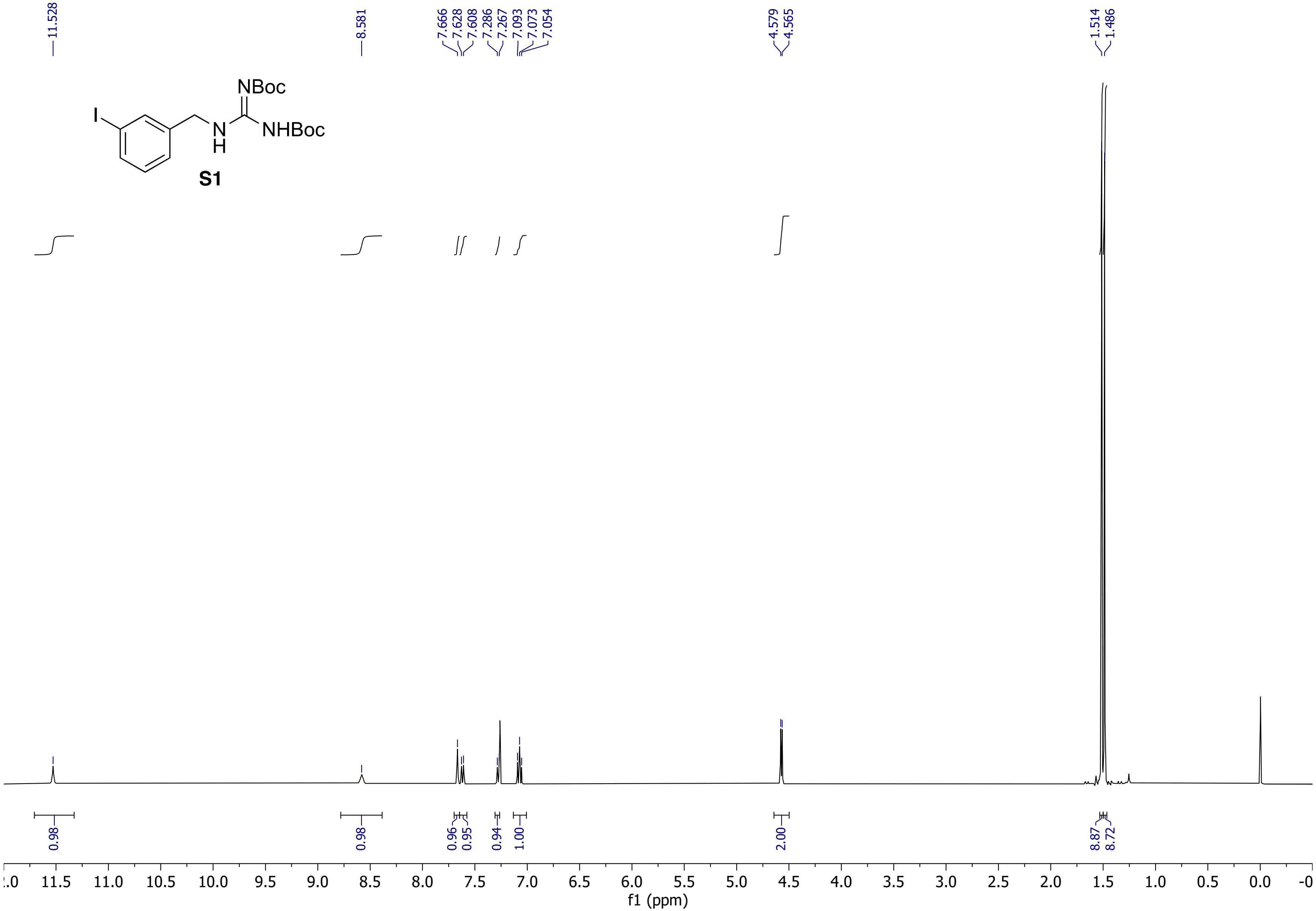

$^{13}C\{^1H\}$ NMR (101 MHz, $CDCl_3$)

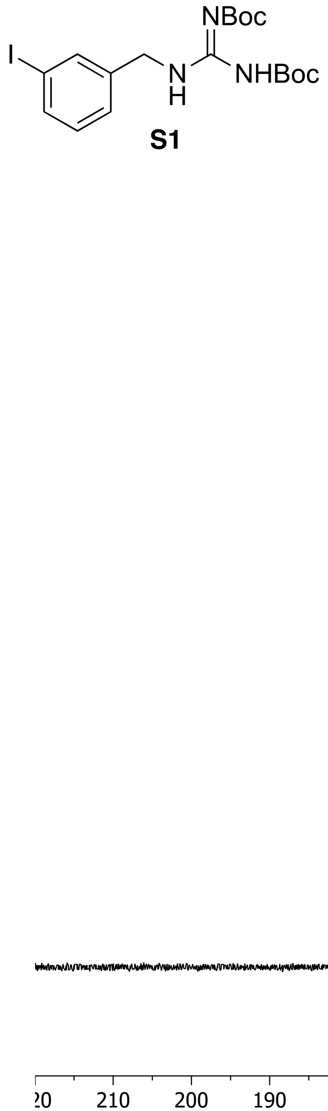

S1

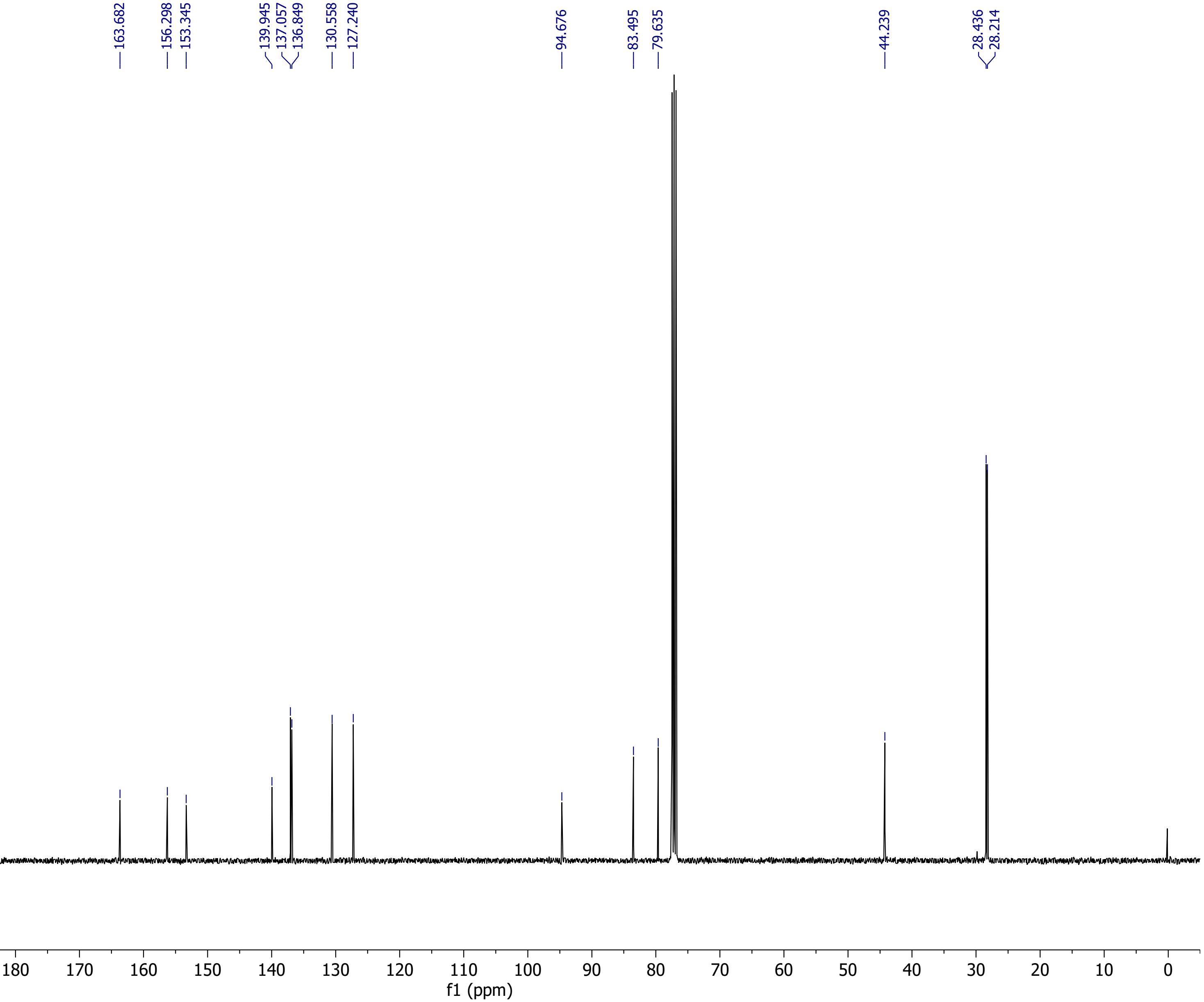

¹H NMR (400 MHz, CD_3OD)

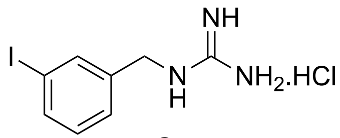

S2

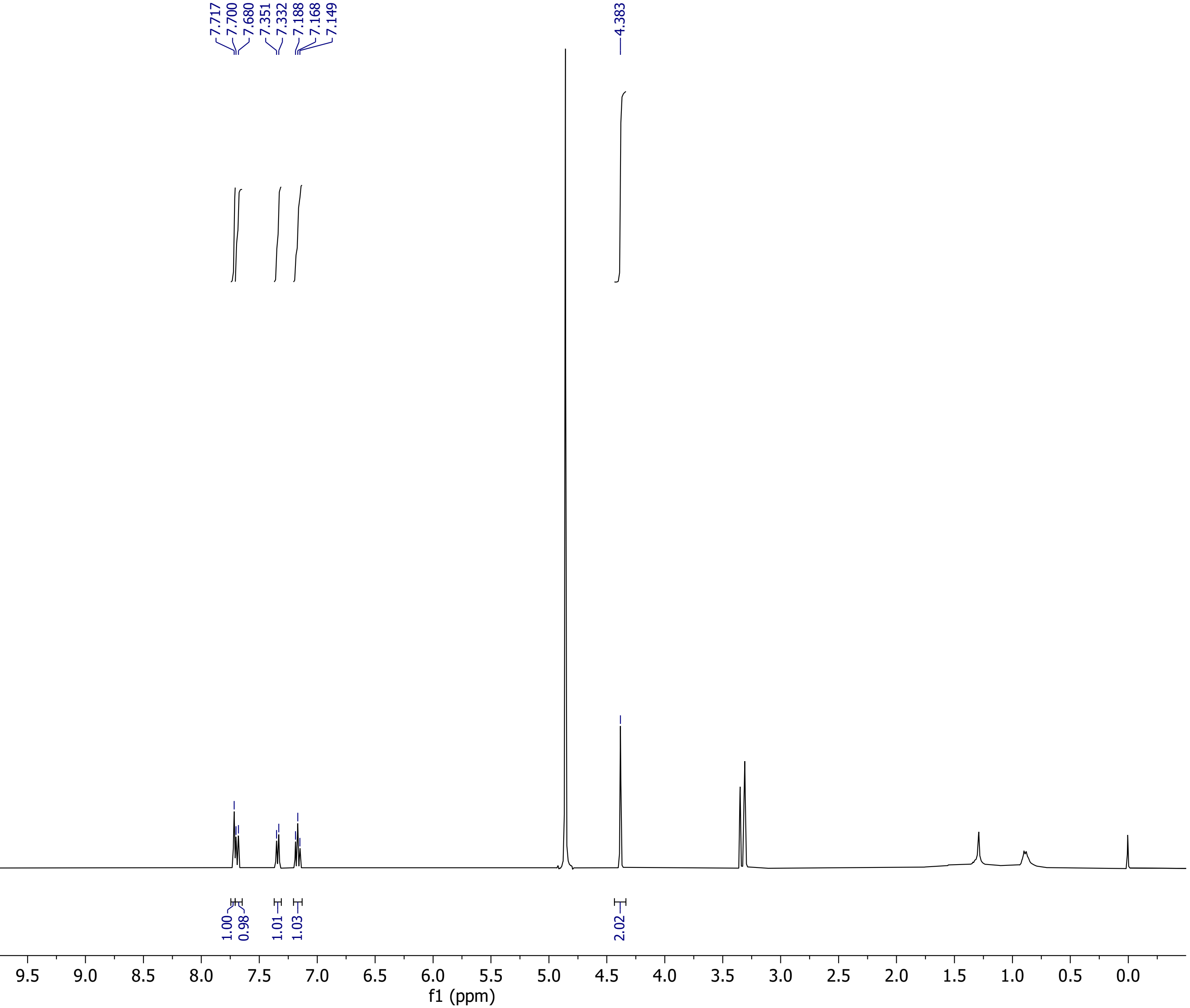

$^{13}C\{^{1}H\}$ NMR (101 MHz, CD_3OD)

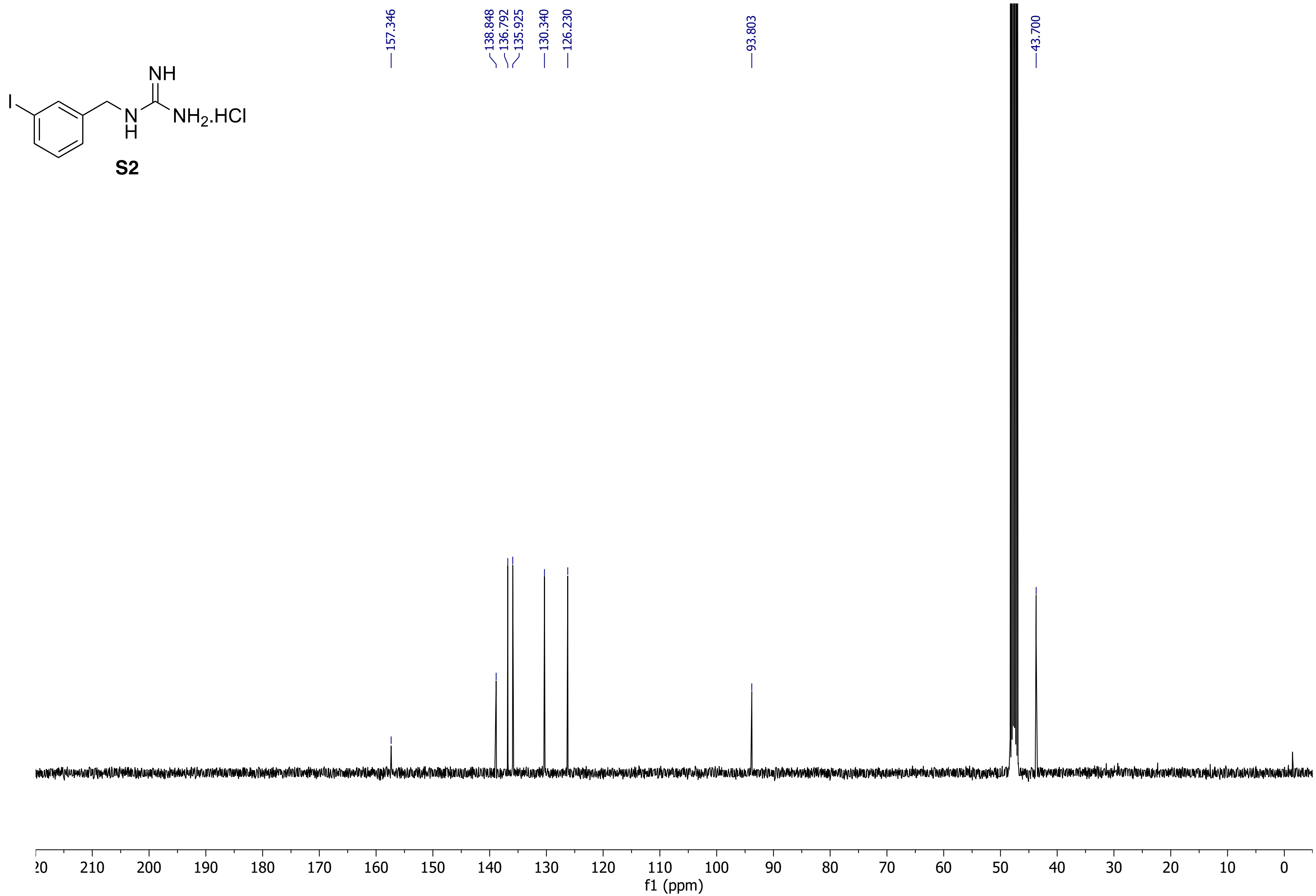

Supplement: Supplementary file 1 — ol4c00356_si_001.pdf [file ol4c00356_si_001.pdf]
